# Comparing a Mixed Model Approach to Traditional Stability Estimators for Mapping Genotype by Environment Interactions and Yield Stability in Soybean [*Glycine max* (L.) Merr.]

**DOI:** 10.3389/fpls.2021.630175

**Published:** 2021-03-31

**Authors:** Mary M. Happ, George L. Graef, Haichuan Wang, Reka Howard, Luis Posadas, David L. Hyten

**Affiliations:** ^1^Department of Agronomy and Horticulture, University of Nebraska-Lincoln, Lincoln, NE, United States; ^2^Department of Statistics, University of Nebraska-Lincoln, Lincoln, NE, United States

**Keywords:** soybean, yield stability, genotype by environment (GxE) interaction, mixed model, association study

## Abstract

Identifying genetic loci associated with yield stability has helped plant breeders and geneticists begin to understand the role and influence of genotype by environment (GxE) interactions in soybean [*Glycine max* (L.) Merr.] productivity, as well as other crops. Quantifying a genotype’s range of performance across testing locations has been developed over decades with dozens of methodologies available. This includes directly modeling GxE interactions as part of an overall model for yield, as well as methods which generate overall yield “stability” values from multi-environment trial data. Correspondence between these methods as it pertains to the outcomes of genome wide association studies (GWAS) has not been well defined. In this study, the GWAS results for yield and yield stability were compared in 213 soybean lines across 11 environments to determine their utility and potential intersection. Both univariate and multivariate conventional stability estimates were considered alongside a mixed model for yield that fit marker by environment interactions as a random effect. One-hundred and six total QTL were discovered across all mapping results, however, genetic loci that were significant in the mixed model for grain yield that fit marker by environment interactions were completely distinct from those that were significant when mapping using traditional stability measures as a phenotype. Furthermore, 73.21% of QTL discovered in the mixed model were determined to cause a crossover interaction effect which cause genotype rank changes between environments. Overall, the QTL discovered via explicitly mapping GxE interactions also explained more yield variance that those QTL associated with differences in traditional stability estimates making their theoretical impact on selection greater. A lack of intersecting results between mapping approaches highlights the importance of examining stability in multiple contexts when attempting to manipulate GxE interactions in soybean.

## Introduction

Establishing a better understanding of the genetic mechanisms which underlie a trait’s variability can lead to greater progress for that phenotype. Grain yield is an example of a trait that displays a complex pattern of quantitative inheritance, dependent on the cumulative action of multiple genes (Falconer, 1996). It has long been recognized that the size and direction of these effects can be influenced differentially by the environmental conditions present over the growing season. These interactions between an individual’s genetics with a wide range of environmental factors are commonly referred to as genotype by environment (GxE) interactions (Comstock and Moll, 1963; Crossa, 1990). This is a crucial consideration as a cultivar will be exposed to a variety of conditions in production settings that cannot be predicted in advance.

With regards to quantitative trait loci (QTL) modeling, plant breeders are often most interested in QTL that result in a consistent effect across environments. GxE interactions present a deviation from this simple additive model, but their contribution to overall phenotype makes them important none the less (Kang, 1997). The evaluation of genotypes across several environments is therefore critical to understanding the contribution of GxE interactions to complex traits such as yield. Due to the contextual nature of GxE interactions, they are often considered a nuisance which obscures the ability to evaluate additive main effects. However, categorizing QTL associated with GxE interactions based on their per environment effects can allow us to highlight those which may be useful for exploitation. Some QTL may have a positive effect on phenotype, but that effect is significantly stronger in some environments. Others are considered “conditionally neutral,” only affecting trait values in some environments but having no effect in others. Both of these sources of GxE variation can have a positive impact on phenotype. Also critical, but less directly useful, are QTL contributing to GxE interactions that have opposing effects in different environments (El-Soda et al., 2014).

The structure of multi-environment trial (MET) data presents several statistical challenges which necessitate a more complex approach to analysis, including those for QTL detection. In a 1952 study, Falconer observed when measuring a trait in different environments that the correlation between those environments was a function of GxE (Falconer, 1952). That is, a high positive correlation is indicative of little to no GxE contribution, while values lower than one revealed GxE as a contributor to the measured trait. Another important consideration of MET data is the influence of GxE interactions on the error variance assumptions. Inherently, GxE interactions often cause the magnitude of genetic variance to differ between individual environments. Explicitly, this means the residual error variance in these analyses often break homogeneity assumptions and failure to account for this has the potential to inflate Type I error rates, especially when those include random GxE interaction terms (Hu et al., 2013, 2014). Assuming genotype [i.e. marker] effects as random in a mixed model approach provides the flexibility to accommodate both differing correlation structures (Piepho, 2005; van Eeuwijk et al., 2010), as well as model a variety of residual error variance structures (Malosetti et al., 2013) and even spatial variation within the error term (Schabenberger and Gotway, 2017). A direct advantage of this analysis structure is the ability to test environment specific QTL effects alongside constitutive main effects, allowing the categorization of QTL into those described above. Additionally, a direct mixed model approach has the advantage of accommodating incomplete and unbalanced datasets that are often common in agronomic field trials (Isik et al., 2017). However, with an increasing number of environments and incorporation of complicated model structures, the number of parameters to be estimated can inflate model size to such an extent that the time and resources to solve it may become impractical (Chen et al., 2010).

Plant breeders aim to select varieties which maintain their high performance across a target region. This trait is commonly referred to as phenotypic “stability,” or sometimes “plasticity.” Differences in stability among genotypes are the natural result of differing GxE interactions (Becker and Léon, 1988). Selecting varieties with superior stability can become difficult when a breeder has to consider all individual GxE interactions and multiple traits for many testing environments. In this case, transforming a multivariate problem such as GxE interactions into a univariate setup is attractive in that it lends itself to more classical analyses styles. Consequently, there has been a long term emphasis on developing methods that can quantify stability into single values that can then be used to rank and compare test genotypes (Lin et al., 1986; Becker and Léon, 1988; Crossa, 1990). Becker and Léon (1988) categorize stability as either static or dynamic. Static phenotypic stability refers to the ability of genotype to produce a consistent phenotype independent from changes in environmental conditions. Dynamic stability describes the genotype’s response to improved agronomic conditions. This is often considered more relevant in production settings where a variety’s ability to respond positively to agronomic inputs such as irrigation and fertilizer is beneficial. However, static stability is often more repeatable and useful for traits such as seed composition which may be expected to meet a certain window of specifications. From the perspective of increasing grain yield, static stability is more relatively advantageous in unfavorable environmental conditions, which is particularly valuable in subsistence agriculture applications (Becker and Léon, 1988).

An increased knowledge of the genetic basis of GxE interactions opens avenues for breeders to manipulate stability through exploiting or minimizing the response to environmental aspects. Several stability measures have recently been used as phenotypes in genome wide association studies (GWAS) to identify novel genomic loci associated with GxE interactions (Bouchet et al., 2016; Xavier et al., 2018; Lozada and Carter, 2020). Explicit mapping of GxE as a marker by environment effect has also been explored, but less considered in stability analyses due to the logistical and computational demands needed to apply the methodology appropriately (Piepho and Pillen, 2004; van Eeuwijk et al., 2010; Malosetti et al., 2013). As yield stability estimates are used to quantify and explain the differences in GxE interactions between genotypes (Becker and Léon, 1988), conducting QTL mapping studies against these values as a phenotype would theoretically reveal some of the same significant loci as directly mapping GxE interactions. A study in barley using both real and simulated data found both static and dynamic stability QTL for several phenotypes that co-located with loci significant in GxE interactions (Lacaze et al., 2009). Similar analyses in tomato reported a lesser degree of intersection, identifying that 24% of the plasticity QTL they discovered were also identified in a mixed model for GxE interactions (Diouf et al., 2020). To our knowledge, this hypothesis has not been tested in soybean population utilizing an unbalanced design for yield trials. Furthermore, past studies have been limited in the number of stability parameters tested in their comparisons. For this study, we report the results of fitting GxE into a mixed model for yield and compare them to using 29 traditional yield stability estimates to map genetic regions responsible for yield stability in a locally adapted soybean population. Yield estimates were obtained for 213 lines grown at five eastern Nebraska sites over three growing seasons. Mapping of yield stability genes was performed both through explicit modeling of marker by environment interactions, and a traditional GWAS approach for conventional stability measures. The potential overlap between identified QTL was investigated with an emphasis on exploring the ability of traditional stability measures to capture the GxE variation present in multi environment yield trials.

## Materials and Methods

### Field Sites and Experimental Design

The University of Nebraska-Lincoln soybean breeding program includes several testing sites across Nebraska but is mostly concentrated in the eastern half of the state where most soybean production occurs. Five testing sites from the breeding program were selected for yield testing that took place over 3 years. Lines belonging to maturity groups I and II were evaluated at the Nebraska locations of Phillips, Cotesfield, and Mead. Lines belonging to group III were evaluated at the Nebraska locations of Phillips, Lincoln, and Wymore (Supplementary Table 1). Yield trials were grown in an augmented incomplete randomized block design at each site, with three replicates per site. Each block consisted of 21-24 entries, with checks assigned according to maturity group. Plots consisted of two rows in 2017, and four rows in 2018 and 2019 to minimize border effects. Rows were 6 meters in length with 0.76 meter spacing between rows. Seeds were sourced from a single location grown in the year prior to that growing season. Prior to planting, seeds were treated with CruiserMaxx at a rate of 1 ml per 200 g, to protect from early season insect and fungal diseases (Syngenta Crop Protection AG, CH-4002, Basel, Switzerland). Grain weight and moisture content were recorded at harvest, and adjusted to 13% moisture to calculate grain yield.

### GWAS Panel Selection and Genotyping

The University of Nebraska-Lincoln soybean breeding program focuses on the improvement of soybean cultivars for producers in eastern Nebraska. Decades of intensive artificial selection through this program has resulted in a collection of genotypes that are highly refined for local conditions. Two-hundred and thirteen experimental lines from the University of Nebraska-Lincoln soybean breeding program were selected to explore and compare mapping methodologies related to GxE interactions across the lines’ target growing region in eastern Nebraska. All lines are F_4_ derived lines created through bi-parental crosses and single seed descent. Lines selected represented a range of both average yield and yield stability from a pool of genotypes that had existing yield data from 2013, 2014, and 2015 multi-environment yield trials. Yield stability was calculated using Wricke’s ecovalence measure, which defines stability as the interaction of the genotype with its environment summed and squared across environments. Therefore, smaller values are considered more stable as they deviate less from the environmental means (Wricke, 1962).

DNA was isolated from lyophilized leaf tissue collected from twenty plants per genotype using a CTAB based extraction method scaled down for a 96 well plate by dividing all reagent volumes by 40 (Keim, 1988). To generate a high density marker panel that enabled a fine mapping resolution while remaining cost effective, whole genome skim sequencing with genotype imputation was used (Happ et al., 2019). The reference panel for imputation was generated from 99 soybean genotypes with publicly available whole genome sequence data, and consisted of 10,803,148 biallelic homozygous single nucleotide polymorphisms (SNPs). Study genotypes were sequenced at a target of < 1X coverage and imputation performed using Beagle 4.1 (Browning and Browning, 2016). All sequence data was deposited in the NCBI Short Read Archive database accession no: PRJNA699266. Pre imputation processing and quality control was performed according to the previously published protocol (Happ et al., 2019). Plink1.9 (Purcell et al., 2007) was used to eliminate individual low quality imputations with a genotype probability (GP) score of less than 0.9. To eliminate redundancy within the SNP panel, 1,129,769 SNPs in close linkage with a pairwise r^2^ value of greater than 0.8 were removed using Plink1.9. Finally, 9,052,059 positions that were non-polymorphic or had a minor allele frequency (MAF) of less 0.05 were filtered out using Plink 1.9. The final genotyping data for the study panel after these steps consisted of 621,320 high quality, homozygous, biallelic SNP markers.

### Accounting for Kinship Between Study Genotypes

Controlling for population structure is an important procedure in association mapping to prevent false positives (Hayes, 2013; Korte and Farlow, 2013). In both scenarios, population structure was controlled through using the first eight principal components in a principal component analysis (PCA) performed in Plink1.9 with a reduced marker dataset. Plink 1.9 first constructs the variance-standardized genetic relationship matrix from marker data before extracting the top 20 principal components (Yang et al., 2011). Markers were first filtered to exclude those with pairwise r^2^ linkage values over 0.4, to prevent the results from capturing linkage disequilibrium patterns. The generated eigenvalues were then visualized as a scree plot to determine the number of principal components to be included in the association mapping analysis (Supplementary Figure 1). As the plot levels off at approximately the eighth component, it was selected for the cutoff. Use of a genomic relatedness matrix to control for confounding relationships was also tested by computing the Balding-Nichols matrix in EMMAX, which estimates the pairwise relationship between individuals using genome wide SNP data (Kang et al., 2010). This was incorporated as a random effect into the described models. Inclusion of this matrix results in a 1.37 and 2.59 point increase in Akaike information criterion (AIC) and Bayesian information criterion (BIC) values, and therefore was dropped from the association analysis as it decreased modeling efficiency with no improvement.

### Association Mapping

Association mapping of both GxE and stability measures required a flexible software that could allow us to fit both linear and mixed models. To this end, we used ASREML-R 4 (Butler et al., 2017) since it provides a wide range of options for modeling both fixed and random effects, as well as the option to include user defined residual error variances structures. Equation (1) below describes the association analyses performed for explicitly mapping GxE by modeling raw yield averaged across replicates with genotype by environmental levels as a per marker random effects:

(1) y=X⁢β+Z⁢α+e

where **y** is the vector of raw yield estimates assumed to be normally distributed, **X** is the design matrix of fixed effects including the intercept, the top eight principal components to control for population structure, environment, and maturity grouping, **β** is the vector of fixed effect coefficients, **Z** is the incidence matrix of random effects including either marker, marker by year, marker by location, or marker by year by location effects, **α** the vector of random effect coefficients, and **e** is the vector of residuals. Allowing for an overall heterogeneous error variance structure resulted in model singularities. Residuals were instead specified as a direct sum of separate variance matrices for each environmental level. Each environmental “level” for the residual is defined as the unique year and location combination. Statistical significance of single markers fit in the linear mixed model was determined using the likelihood ratio test (LRT). This compares the log-likelihood of the model including the marker effect with the log-likelihood of the model without the marker effect. A multiple testing correction was applied via a Bonferroni threshold (α = 0.05) to define significant associations. Results were plotted in a Manhattan plot of –log10 *p*-values using R3.6 (R Core Team, 2019) with package “ggplot2” (Villanueva et al., 2016).

A wide variety of approaches for calculating yield stability pervades across scientific literature. Recently, Pour-Aboughadareh et al. (2019) reported the development of an R script to calculate a range of phenotypic stability estimates, providing a manageable way to calculate sixteen popular stability estimates using a single R function. This included Plaisted and Peterson’s mean variance component, Plaisted’s GE variance component, Wricke’s ecovalence stability index, regression coefficient, deviation from regression, Shukla’s stability variance, environmental coefficient of variance, Nassar and Huhn’s statistics (S1 and S2), Huhn’s equation (S3 and S6), Thennarasu’s non-parametric statistics (NP1-4), and Kang’s rank-sum (Happ et al., 2019). While this covered many of the prevalent univariate stability analysis methods, it did not include the multivariate additive main effect and multiplicative interaction (AMMI) analyses methods and subsequent stability values (Sabaghnia et al., 2008). AMMI modeling has been widely used in plant breeding programs to investigate GxE interactions and provide stability estimates through first isolating GxE interactions using a linear model that accounts for some of the main experimental design effects (Abera et al., 2004; Ezatollah et al., 2011; de Oliveira et al., 2014). An AMMI analysis was subsequently performed with the raw yield data and thirteen stability estimates calculated in R3.6 using package “ammistability” (Ajay et al., 2018), including the sum across environments of genotype by environment interactions (GEI) modeled by AMMI (AMGE), AMMI stability index (ASI), AMMI stability value (ASV), AMMI based stability parameter (ASTAB), sum across environments of absolute value of GEI modeled by AMMI (AVAMGE), Annicchiarico’s D parameter (DA), Zhang’s D parameter (DZ), averages of the squared eigenvector values (EV), stability measure based on fitted AMMI model (FA), modified AMMI stability index (MASI), modified AMMI stability value (MASV), sums of the absolute value of the IPC scores (SIPC), absolute value of the relative contribution of IPCs to the interaction (Za). Association mapping with each of these stability measurements was also performed in ASREML-R 4 per SNP marker following a typical linear model listed in equation (2) below:

(2) y=X⁢β+e

where **y** is the vector of one of the yield stability estimates assumed to be normally distributed, **X** is the design matrix of fixed effects including the intercept, the top eight principal components to control for population structure, and the individual marker being tested, **β** is the vector of fixed effect coefficients, and **e** is the vector of residuals. The model assumes that $e∼N\,\left(0,I\,\sigma_{e}^{2}\right)$. Fitting this model using Nassar and Huhn’s S2 statistic, statistical significance of single markers fit in the linear mixed model was determined using the Wald test procedure that is part of the ASREML-R 4 package. A multiple testing correction was applied via a Bonferroni threshold (α = 0.05) to define significant associations. Results were plotted in a Manhattan plot of –log10 *p*-values using R3.6 and package “ggplot2.”

### Overlap and GxE Variance Explained by QTL

If QTL, via association mapping with yield stability as a phenotype, captures genomic regions involved in GxE interactions, we would expect to see some degree of overlap with QTL identified in the explicit GxE association mapping. To visualize this, the bounds of significant QTL from each association model broadly classified as either GxE, multivariate conventional (AMMI), or univariate conventional were plotted using R3.6 and package “karyoploteR” from Bioconductor (Bernat and Serra, 2017). These were color coded according to model classification. Overlaps between QTL from each model classification were also plotted as a Venn Diagram using R3.6 with package “VennDiagram” (Chen and Boutros, 2011).

Contribution and impact of QTL can be characterized by computing their contribution to overall trait variance. If QTL discovered via association mapping with yield stability as a phenotype captures genomic regions involved in GxE interactions, it could be assumed that these regions would explain significant portions of GxE variance for yield. For each of the methods described, we computed the proportion of yield variance explained by GxE for the most significant SNP, that is, the SNP with the lowest *p*-value, in each individual QTL region. After fitting equation (1) in ASREML-R 4, variance component estimates from the random effects’ solutions were extracted for each marker and proportion of yield variance explained by GxE calculated using equation (3) below:

(3) $$\frac{m\,a\,r\,k\,e\,r\times e\,n\,v\,i\,r\,o\,n\,m\,e\,n\,t}{g\,e\,n\,o\,t\,y\,p\,e+e\,n\,v\,i\,r\,o\,n\,m\,e\,n\,t+\left(m\,a\,r\,k\,e\,r*e\,n\,v\,i\,r\,o\,n\,m\,e\,n\,t\right)+r\,e\,s\,i\,d\,u\,a\,l}$$

For each model, the average and standard deviation of these values from all QTL was calculated. The results were plotted in R3.6 using ggplot2 and color coded according to model classification.

### GxE Interaction Type

GxE interactions create noise in multi environment trials that make it difficult to identify which genotypes are superior. The two potential outcomes are changes in genotype ranking or a change in distance between rankings. To categorize the effect of each of the QTL discovered via direct GxE modeling, we extracted the effect size of each allelic state at individual environmental combinations for comparison. Effects larger than 6.8 kg/ha (0.25 bu/a) were significant at an alpha value of 0.05 and thus were the only effects considered for this analysis. If all effects were in one sign (all positive or all negative), the QTL was classified as a magnitude interaction. If one of more effects were of an opposite sign than the others, the QTL was considered a crossover interaction. This was performed for all 56 GxE QTL. At each QTL we also examined the overall and per environment adjusted yield distributions. Results of each were plotted in R3.6 using ggplot2.

### Principal Component Analysis of Rankings

Plant breeders are often interested in ranking genotypes to make advancement selections. We compared the rankings from yield stability measurements to those ascertained from the Best Linear Unbiased Predictor (BLUP) of the various GxE interactions levels. BLUPs were calculated in ASREML-R 4 according to equation (1), where the incidence matrix **Z** instead included the random effects of genotype, genotype by year, genotype by location, and genotype by year by location effects. To rank genotypes via these BLUPs, the absolute value of the BLUP values were taken and then ordered from smallest to largest. Therefore, the smallest GxE BLUP value denoted the most “stable” genotype. Rankings for the conventional yield stability measures were assigned according to their definition. In all cases, a ranking of “1” denoted the most stable genotype. A principal component analysis of these rankings was conducted in R3.6 using the “prcomp” function which is a part of basic R functionality. Results from the principal component analysis were plotted using the “ggplot2” package and color coded according to model classification.

## Results

### Phenotype and Genotype Data

The 213 soybean experimental lines were yield tested in an augmented incomplete randomized block design at five eastern Nebraska locations over 3 years to assess grain yield stability. Grain yield over the course of these trials ranged from 2162.74 to 7080.70 kg/ha, with an average of 4976.41 kg/ha and standard deviation of 810.34 kg/ha. The highest yielding year was 2017 with an average grain yield of 5204.80 kg/ha and highest yielding location was Phillips, which averaged 5570.40 kg/ha (Supplementary Table 2). Distribution of yield values were approximately normal when examined visually per environment (Supplementary Figure 2). Likewise, the association panel captured a wide range of stability values both in the univariate and multivariate measures (Supplementary Tables 3, 4). Additionally, correlations between univariate stability parameters were much lower than the multivariate stability parameters computed for this study. This suggests capture of different aspects of stability and GxE interactions with the exception of perfect correlations between Wricke’s ecovalence, Shukla’s stability variance, the GE variance component, and the mean variance component (Supplementary Figure 3).

Construction of genotype information was performed using low coverage whole genome sequence data with imputation using a reference panel of deep sequenced soybean genotypes. DNA extracted from leaf tissue collected in 2016 from the study genotypes was used to perform whole genome sequencing at a minimum coverage of 0.3X. After post imputation quality control, the final genotyping panel consisted of 621,320 high quality, homozygous, biallelic SNPs with 1.79% of marker genotypes missing. Per marker missing data rates ranging from 0.34 to 7.74% with a standard deviation of 0.86%.

### Association Mapping

**FIGURE 1:**
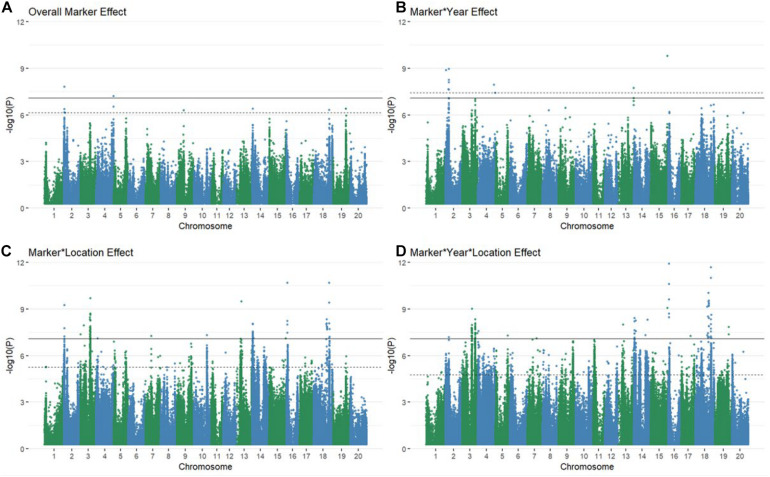
Manhattan plots of marker **(A)** and marker by environment **(B–D)** levels modeled explicitly as random explanatory variables of raw grain yield. Several associations are significant at every level via both the Bonferroni correction (solid black line) and a 5% FDR (dashed line) with some overlap between QTL discovered for varying levels of GxE interactions **(B–D)**.

Using multiple approaches to map genetic loci associated with grain yield stability in the 213 genotypes revealed 106 significant QTL via the Bonferroni threshold. 86 of these were determined to be independent between all mapping approaches when considering overlaps between QTL bounds (Supplementary Figures 4A–32A and Figure 1). The majority of QTL associated with GxE interactions were found in the marker^∗^location and marker^∗^year^∗^location terms, with some degree of overlap between all interactive terms (Figures 1B–D). The number of QTL for overall yield was affected by inclusion of environmental interaction terms, resulting in one additional QTL significant via the Bonferroni threshold and shifting which QTL were significant at a FDR of 5% (Figure 1A and Supplementary Figure 4A). Models fitting the coefficient of variation, Finlay Wilkinson, Sum Across Environments of Absolute Value of GEI Modeled by AMMI, and Zhang’s D Parameter as phenotypes did not return any associations that were significant by either the Bonferroni correction or a FDR of 5%. The AMMI stability value and AMMI stability index only returned associations that were significant using a 5% FDR threshold. Model inflation was assessed by examining the quantile-quantile plots of *p*-values produced by each model fit (Supplementary Figures 4B–32B, 33). Deviation from the diagonal suggested considerable inflation in models fitting the GE variance component, mean variance component, Shukla’s stability variance, Thennarasu NP2 statistic, and Wricke’s ecovalence, and were therefore dropped from consideration in further analyses.

**FIGURE 2:**
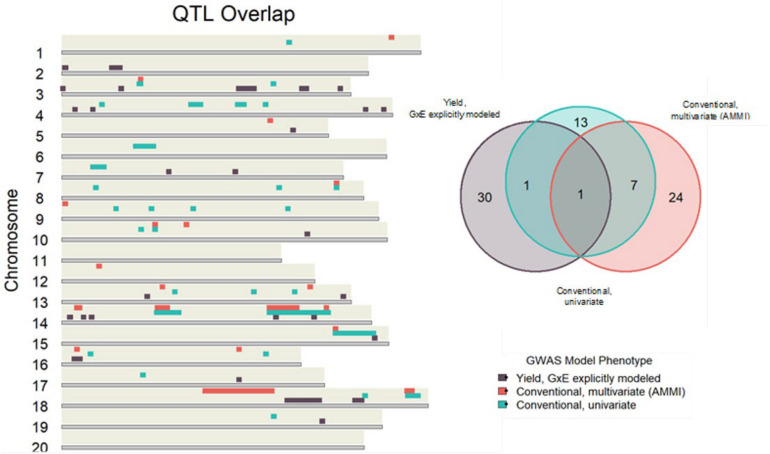
Independent QTL discovered using conventional measures as a GWAS phenotype share very little overlap with loci significant in the explicit GxE model.

**FIGURE 3:**
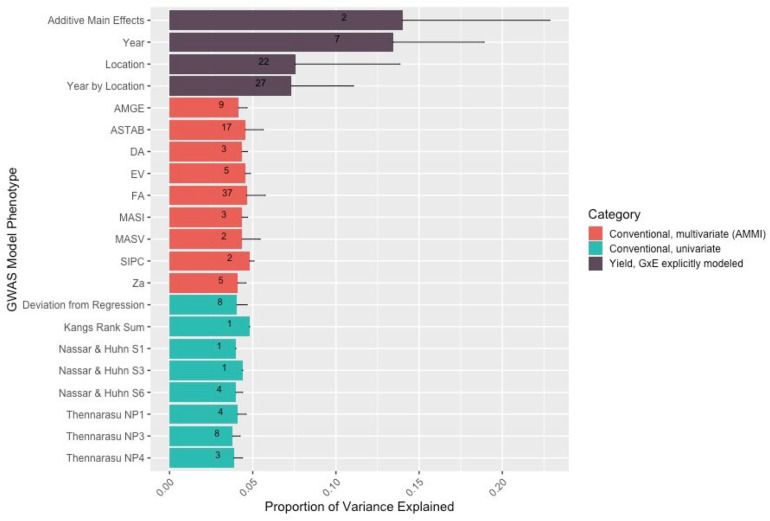
The number and variance explained by the QTL discovered in the explicit GxE model is greater than that discovered by GWAS models using either type of conventional measurement as a phenotype. Numbers within the bars represent the number of QTL discovered for that model/model level. The thin dark line from the top of the bar represents the standard deviation for yield variation explained among the QTL for that level.

Conventional yield stability measures are assumed to explain genotype differences in GxE interactions of multi-environment trials and distill them into a singular value. Overlap between QTL discovered using conventional measures as a phenotype and explicitly modeling GxE interactions may indicate the extent of their interchangeability. Considering the boundaries of the 86 independent QTL discovered in the association mapping, we found only one QTL shared among all three modeling approaches. Univariate and multivariate approaches shared eight intersecting QTL with each other, but only two and one QTL with explicit GxE modeling, respectively (Figure 2). Comparing the average yield variance explained by GxE at each of the QTL among approaches revealed that significant loci as reported by the explicit GxE model accounted for more GxE yield variance than either conventional approach. The largest number of QTL were discovered for the marker by year by location, and marker by location interaction effects, however the average effect size was lower than those QTL associated with additive main effects and genotype by year interactions (Figure 3). These results suggest that using conventional yield stability estimates as a phenotype for GWAS is not a substitute for directly modeling GxE interactions.

### Classification of GxE Interactions

**FIGURE 4:**
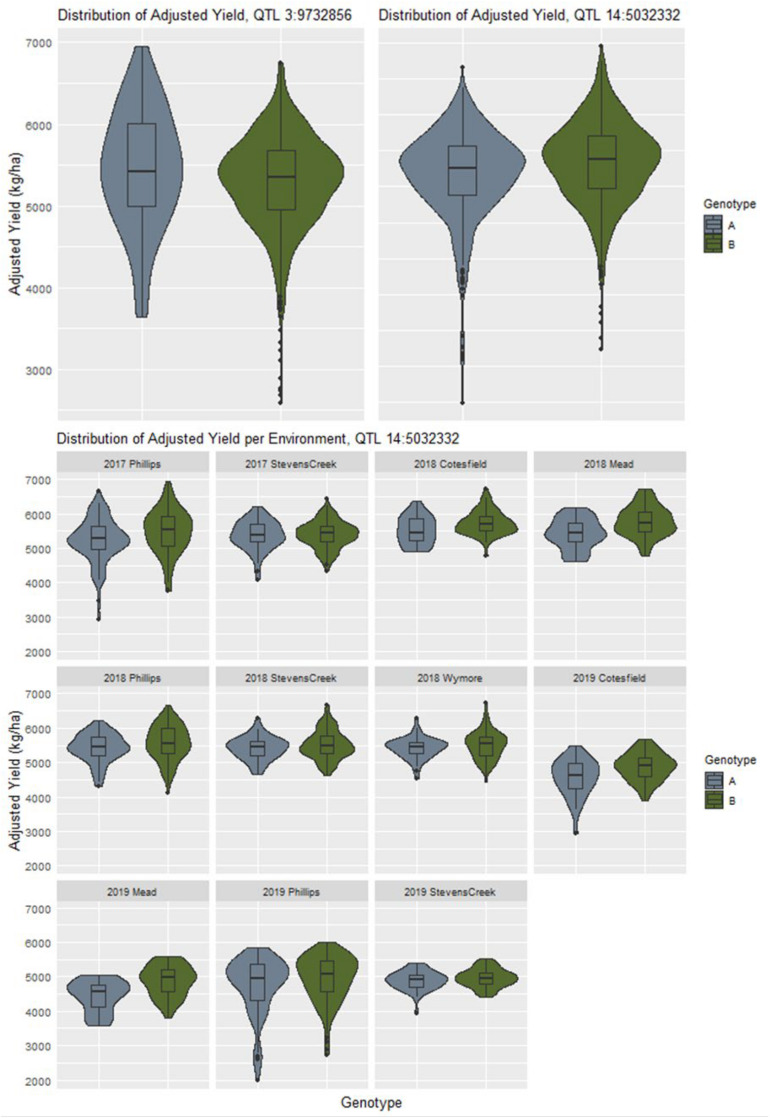
The contrast in distribution of adjusted yield between allelic states at the QTL on chromosome 3 indicates a difference yield stability as compared to the QTL on chromosome 14 which initially appears to be falsely associated. However, when examining the adjusted yield from a per environment basis, differences in mean and spread according to specific site combinations become more apparent.

If a locus is involved in creating changes in yield stability, it can often be seen as a difference in dispersion between the phenotypic distributions between alleles (Rönnegård and Valdar, 2012). The distributions of adjusted phenotypes according to allele states at the QTL discovered by explicitly modeling GxE interactions for yield did not appear to follow this trend, with many of the distributions appearing to be nearly identical (Supplementary Figure 34). For example, the QTL at chromosome 3 physical position 9732856 shows a more characteristic difference in adjusted phenotype distributions that the QTL at chromosome 14 physical position 5032332 (Figure 4). Genotypes with allele “A” at the former QTL are less stable, as indicated by the flatter and wider distribution of adjusted phenotypes. When looking at all the adjusted phenotypes pooled across environments for QTL 14:5032332, we do not see an initial difference in distributions despite it being reported as a GxE QTL by the model. Upon examining the same data on a per environment basis, we see contrasts in mean and dispersion that are not consistent across year and location combinations (Figure 4). This is an indication of a crossover interaction occurring at this locus which has a canceling effect when assessing data combined across environments.

**FIGURE 5:**
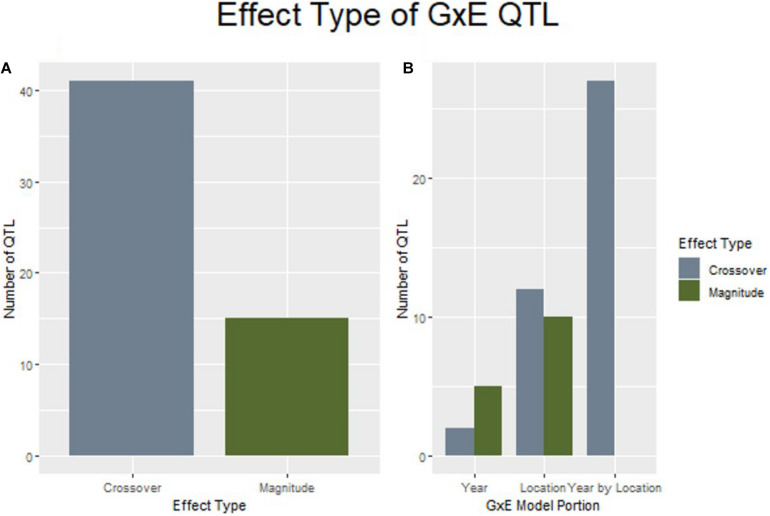
QTL of the crossover effect type are more prevalent in this study than magnitude changes **(A)**, and are espeically common in the marker by year by location interaction **(B)**.

GxE interactions complicate the breeding process the most when they result in a crossover interaction that changes genotype rankings between growing environments. When examining the effect size and direction of QTL discovered by explicitly modeling GxE interactions for yield, 41 of the 56 loci were considered to produce crossover interactions. Further, it was noted that all QTL discovered through modeling marker by year by location were determined to be crossover interactions (Figure 5).

### Selection Rankings

**FIGURE 6:**
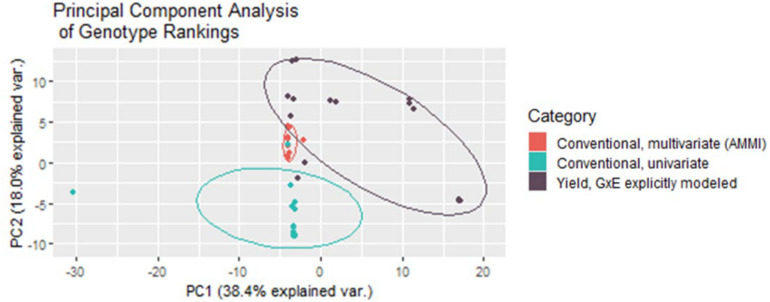
Multivariate conventional yield stability rankings group much tighter and closer to rankings generated from the BLUPs from fitting GxE interaction effects as random in the mixed model for yield.

Both multivariate and univariate conventional yield stability measures are used to create rankings that help breeders make selection decisions. Conducting a principal component analysis of these rankings in comparison to the rankings given by the BLUPs of the direct GxE model showed that multivariate conventional measures generated from AMMI modeling grouped very tightly together, and the closest with GxE BLUP rankings. Univariate yield stability measures also generally grouped together, intersecting far less with the GxE groupings than multivariate statistics (Figure 6). Together the first two principal components explained 56.4% of variability in the rankings indicating that these groupings still do not capture nearly half of the data variance.

## Discussion

This analysis revealed that using conventional stability estimates to capture variation in GxE interactions for a genetic mapping study gave considerably different results from directly modeling GxE interactions when evaluating grain yield in a local soybean population. GxE interactions often heavily influence the per environment rankings of quantitative phenotypes such as grain yield, complicating the breeders’ task of developing a stable variety. As a result, the modeling of phenotypic stability and identification of the involved genes has been the focus of many recent scientific studies (Bouchet et al., 2016; Xavier et al., 2018; Lozada and Carter, 2020). QTL affecting stability have been discovered using either a direct approach to modeling GxE interactions, or first calculating a yield stability “value” from the phenotypic data to then be used a phenotype in the GWAS. This study evaluates both of these approaches using the same association panel and demonstrated that the results were not interchangeable. To our knowledge this is the first study to directly compare QTL discovered using multiple methods of evaluating GxE interactions and yield stability in soybean.

Conventional yield stability estimates are a popular way to assess the influence GxE interactions without the burden of interpreting values for every testing environment. Such approaches are both appealing from a computational standpoint due to their simplicity, as well as pragmatic when discussing plasticity and stability within the scientific community. The historical implementations of traditional stability methods presented here are built upon variations on standard linear regressions – that is, a statistical model that only has fixed effects. When considering datasets from multi-environment trials with missing observations, the results may be to varying extents, erroneous (Piepho, 1997). This is one potential rationale for the large difference in QTL results presented by this study compared to present research. However, unbalanced experimental designs are common in plant breeding programs both due to random loss within trials (pest/disease/weather damage/etc.) or by explicit design, and their accommodation should be prioritized. Mixed model analyses have been an effective tool in this regard. Recent studies have shown they can also be used to adapt traditional stability analyses, suggesting that conventional yield stability estimates can still provide useful insights from more complex field designs (Piepho, 1997, 1998; Meyer, 2009). Explicitly testing the consequences of using conventional yield stability statistics on unbalanced datasets may help refine the understanding of these results and future applications.

QTL confirmation testing would be an important step to validating the modeling approach used in their discovery. Due to unpredictable fluctuations in environment, the results of GxE interaction research are often difficult to replicate. With regards to QTL studies, this adds difficulty to the confirmation process. If effective, the results might not only be used to support the existence of a QTL affecting GxE interactions, but also serve to refine the understanding of what general effect it causes. This may be especially important when considering antagonistic crossover effects, which have the largest confounding effect on making selections in the plant breeding process. The results presented here indicate that the greater majority of GxE interactions in grain yield for soybean generate a crossover effect, with neither observed allele advantageous in all testing environments. In fact, in some cases opposite effects were nearly equal and appeared to cancel each other out when observing the pooled data. Extensive testing of GxE QTL in new genetic backgrounds and new environments may reveal a shift in these QTL classifications and ultimately their utility to breeders. Detecting and accounting for crossover effects may be important to breeding decisions for local adaptations as well as separating out these noisy interactions from those that are more straightforward to incorporate.

Plant breeders often use a ranking approach to eliminate or progress genotypes in their breeding program (Sjoberg et al., 2020). Similarly, to the GWAS results, comparing the genotype rankings from each of the methods demonstrated the dissimilarity between conventional and explicit GxE modeling approaches. A multivariate approach (AMMI) which first starts with a model that retains some of the experimental design components best match the rankings from the GxE BLUPs themselves, further illustrating the importance of accounting for this additional variation in this experiment. The rigidity of software created for the purpose of computing traditional stability estimates limits the inclusion of non-genetic design components, such as replicate, location, blocking, and/or other spatial factors that may have been valuable in partitioning genetic variance from the phenotypic variance. Without prior adjustment, these artifacts have the potential to bias results and decrease selection accuracy for phenotypic stability.

## Conclusion

This analysis determined that performing association mapping for grain yield GxE interactions in soybean using conventional yield stability measurement as a phenotype provided nearly independent results from explicitly modeling marker by environment interactions in a mixed model for grain yield. While several QTL were discovered using both approaches, only one region overlapped between models and QTL discovered via conventional stability estimates explained far less GxE variance for grain yield. The results presented may have been influenced by the incomplete and unbalanced data structure utilized in the multi-environment trials, however this is a common occurrence in field trials and is often intentional to sample more genotypes and environments. Researchers and breeders interested in manipulating adaptation via GxE interactions need to consider the potential influences their modeling approach will have on their desired outcome.

## Data Availability Statement

The datasets presented in this study can be found in online repositories. The names of the repository/repositories and accession number(s) can be found below: NCBI BioProject, PRJNA699266; European Variation Archive, Project: PRJEB43548 and Analyses: ERZ1756748.

## Author Contributions

MH: methodology, software, formal analysis, investigation, writing—original draft, and visualization. GG: conceptualization, resources, writing—review and editing, supervision, project administration, and funding acquisition. HW: investigation. LP: methodology, software, and writing—review and editing. RH: methodology and writing—review and editing. DH: conceptualization, methodology, resources, writing—review and editing, supervision, project administration, and funding acquisition. All authors contributed to the article and approved the submitted version.

## Conflict of Interest

The authors declare that the research was conducted in the absence of any commercial or financial relationships that could be construed as a potential conflict of interest.
