# Supplementary material for: Comparing a Mixed Model Approach to Traditional Stability Estimators for Mapping Genotype by Environment Interactions and Yield Stability in Soybean [Glycine max (L.) Merr.]
Source: Front Plant Sci. 2021 Mar 31;12:630175. doi: 10.3389/fpls.2021.630175 (PMC8044453; doi:10.3389/fpls.2021.630175)
Supplement: Supplementary file 1 [file Data_Sheet_1.docx]

**
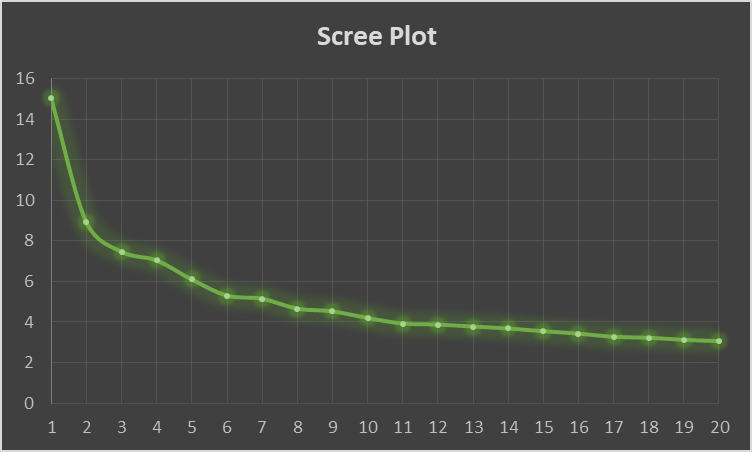
**

Supplementary Figure 1: The trend line drawn between eigenvalues from a principal component analysis of the genotypic dataset shows a reduction in slope for subsequent points around the eighth principal component. Thus, the first eight principal components were used in our GWAS analysis.


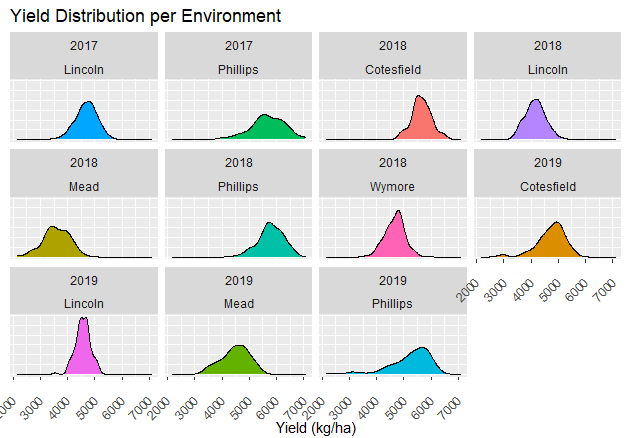


Supplementary Figure 2: Yield distribution is approximately normal per year and location combination.


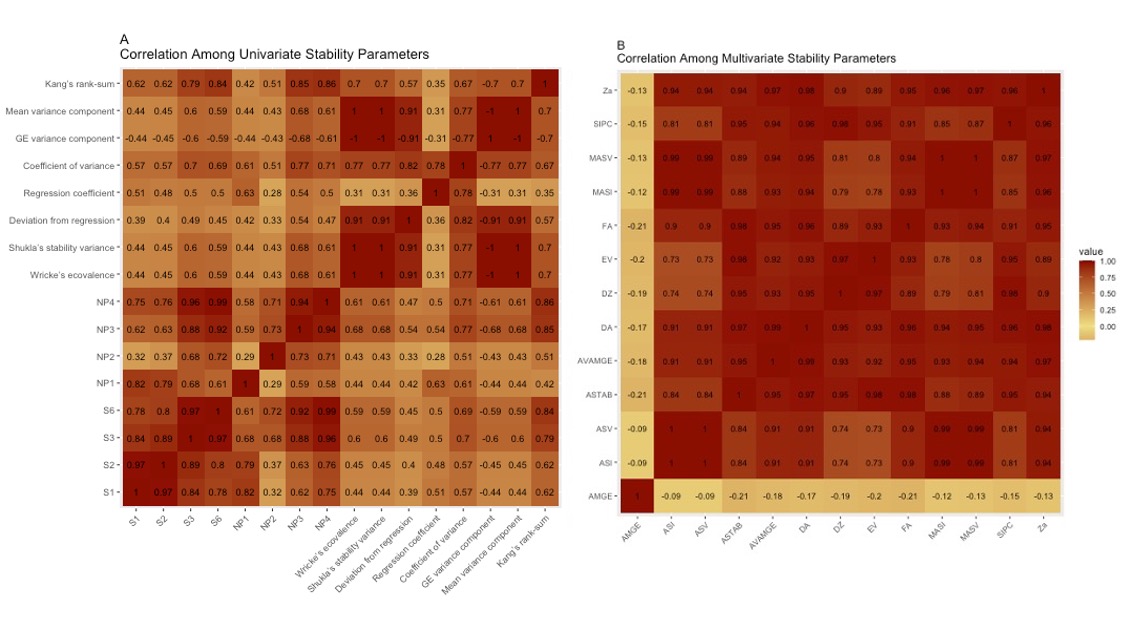


Supplementary Figure 3: Correlation matrices for traditional stability statistics reveals that while many of the multivariate stability parameters are highly correlated with each other, many of the univariate measures are fairly distinct. Notably, Wricke’s Ecovalence, Shukla’s Stability Variance, the GE variance component and mean variance components were all perfectly correlated with values of 1 or -1.


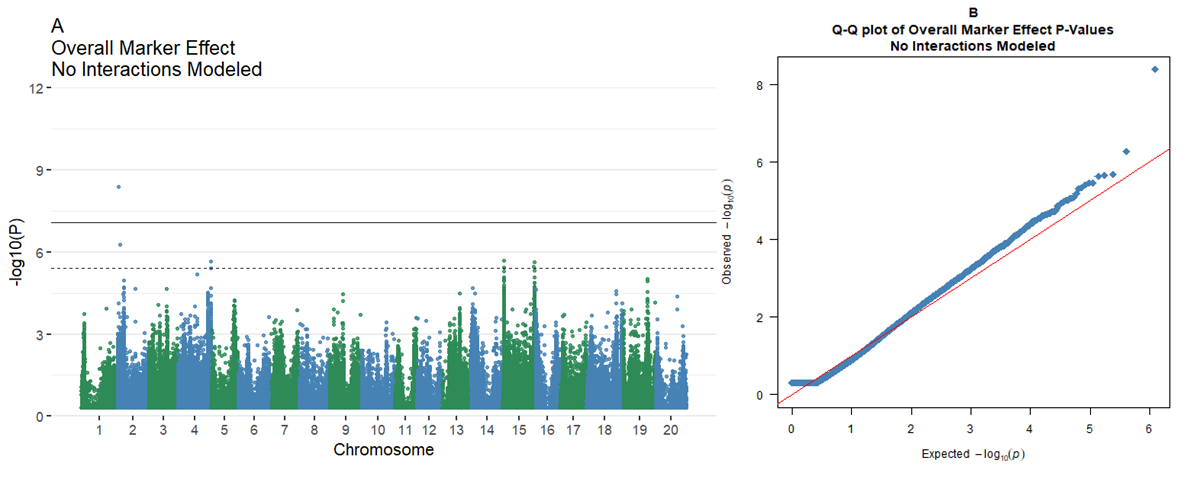


Supplementary Figure 4: Manhattan plot (A) and q-q plot (B) of the GWAS results for yield without fitting genotype by environment interactions 1 QTL is significant via Bonferroni correction, and an additional 3 QTL are significant when considering a FDR of 5%.

**
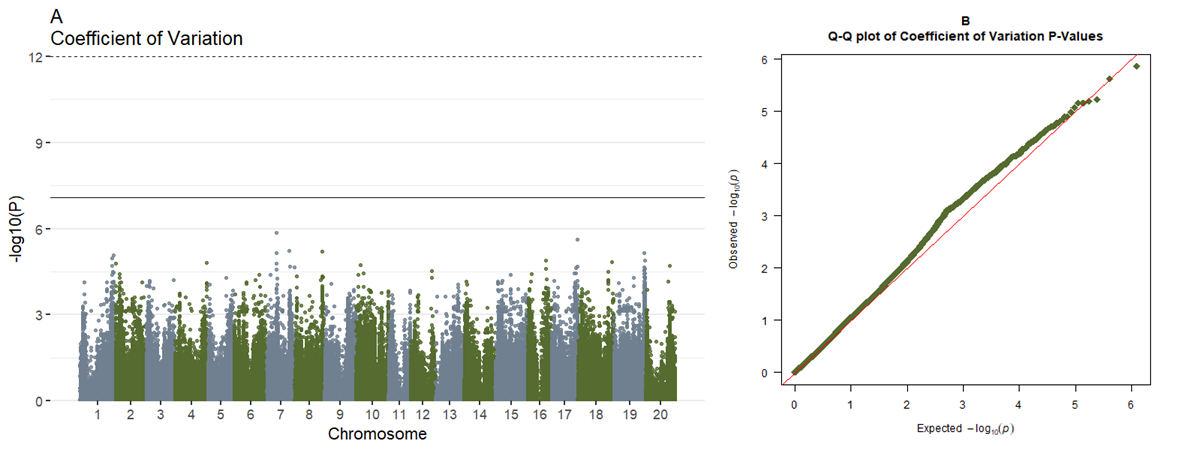
**

Supplementary Figure 5: Manhattan plot (A) and q-q plot (B) of GWAS results using the coefficient of variation as the model phenotype.


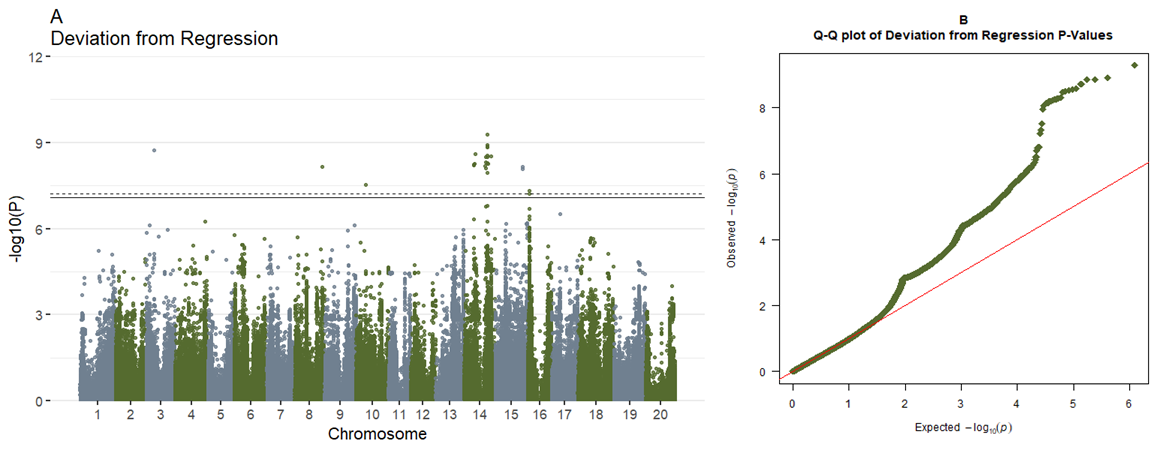


Supplementary Figure 6: Manhattan plot (A) and q-q plot (B) of GWAS results using the deviation from regression as the model phenotype.


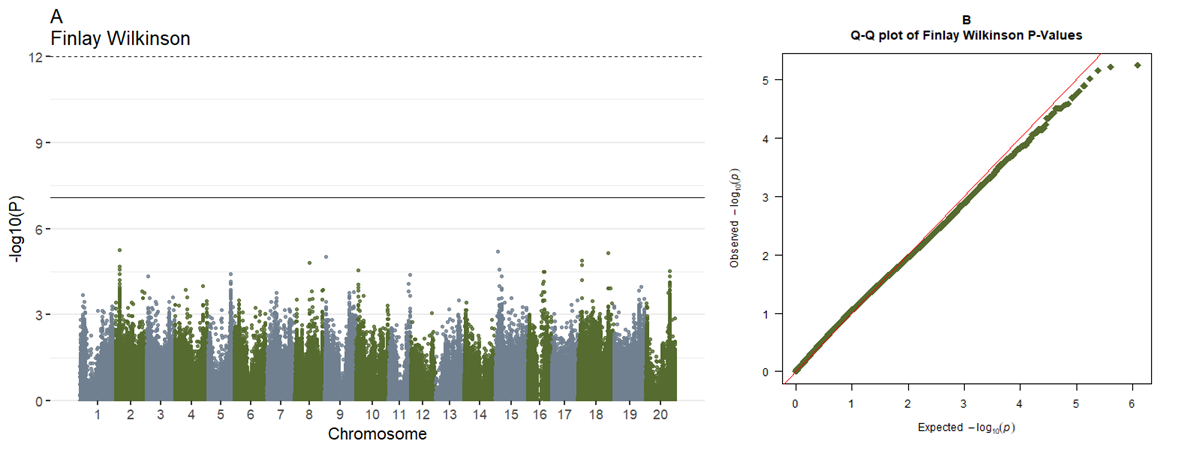


Supplementary Figure 7: Manhattan plot (A) and q-q plot (B) of GWAS results using the Finlay Wilkinson value as the model phenotype.


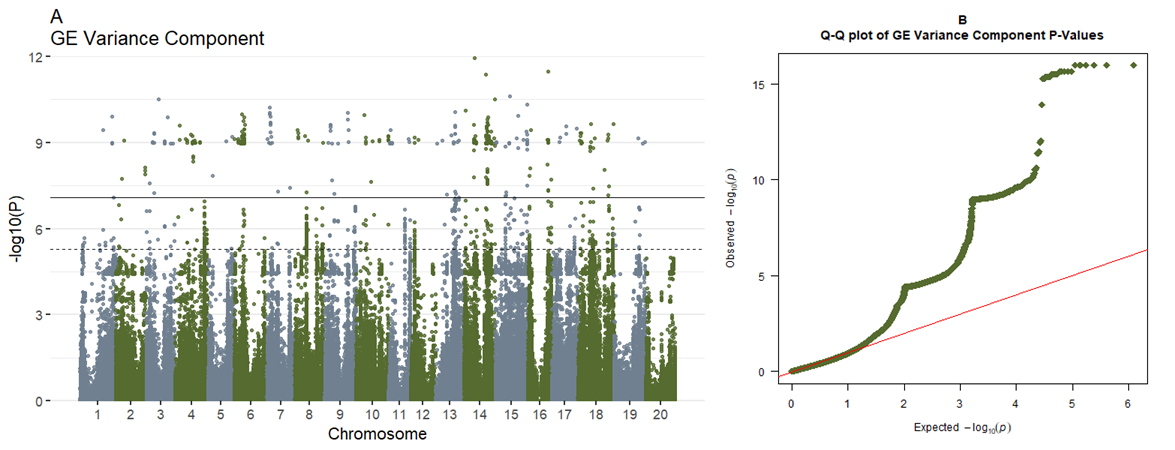


Supplementary Figure 8: Manhattan plot (A) and q-q plot (B) of GWAS results using the GE variance component as the model phenotype.


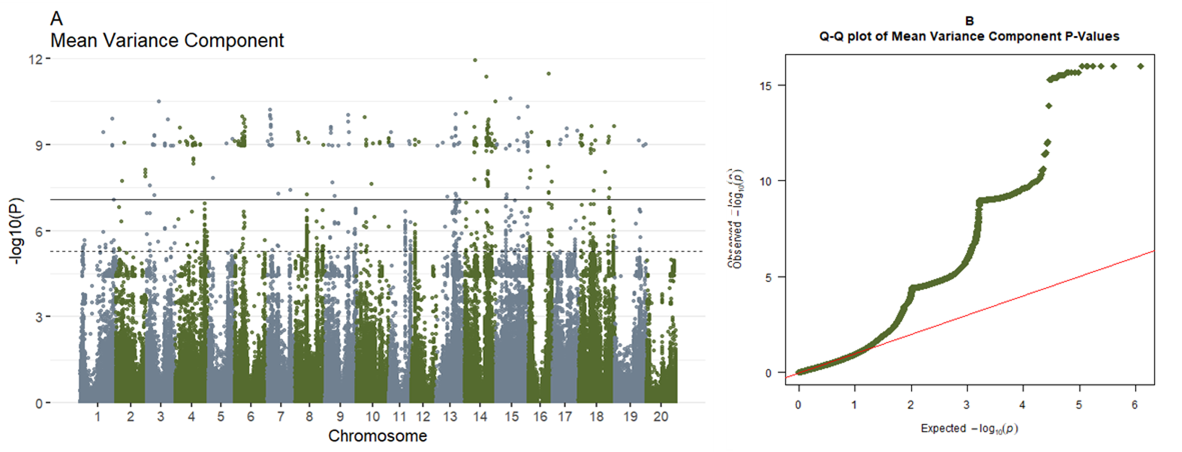


Supplementary Figure 9: Manhattan plot (A) and q-q plot (B) of GWAS results using the mean variance component as the model phenotype.


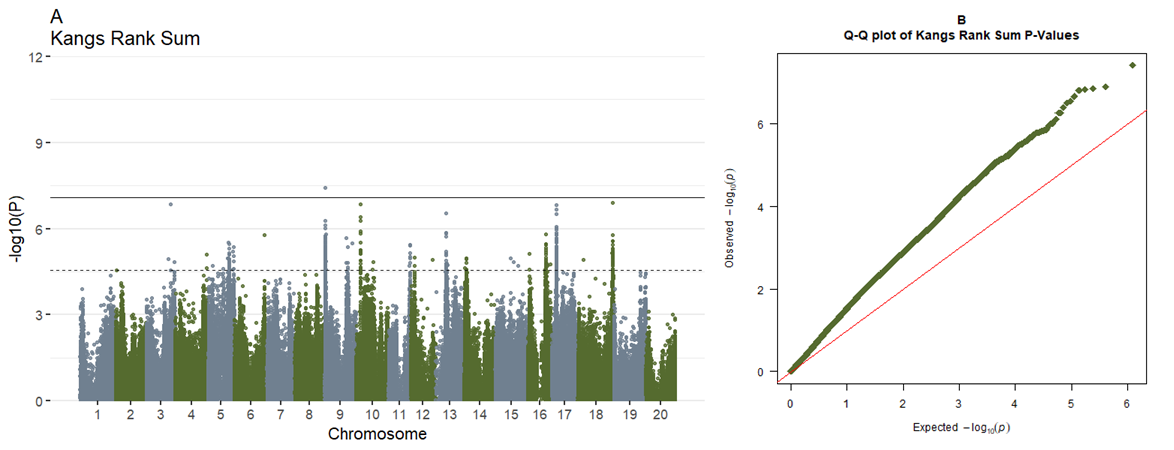


Supplementary Figure 10: Manhattan plot (A) and q-q plot (B) of GWAS results using Kangs Rank Sum as the model phenotype.


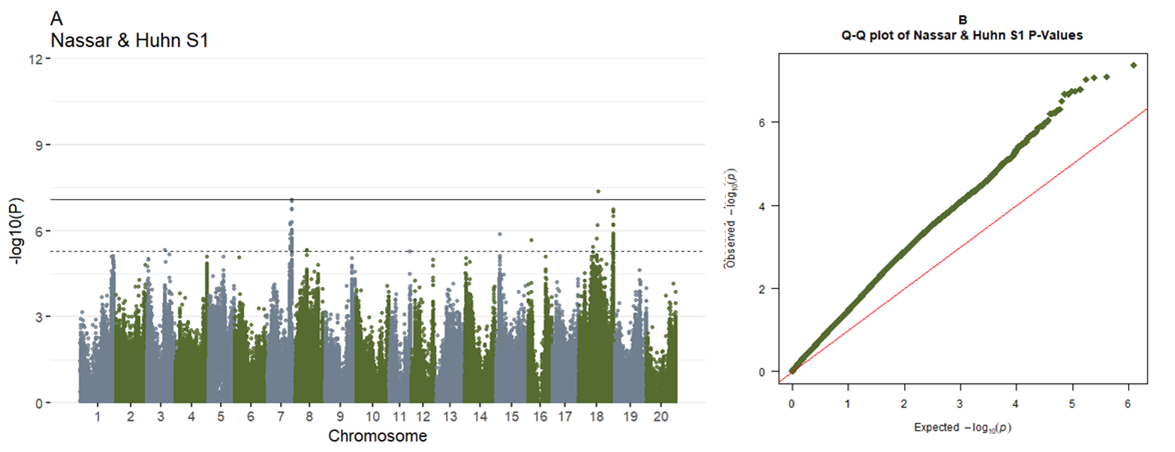


Supplementary Figure 11: Manhattan plot (A) and q-q plot (B) of GWAS results using the Nassar and Huhn S1 statistic as the model phenotype.


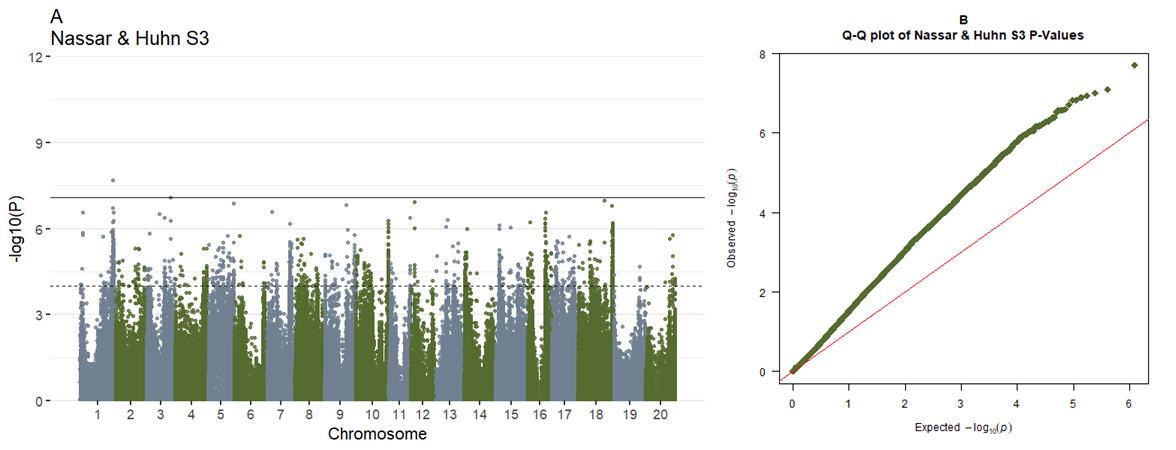


Supplementary Figure 12: Manhattan plot (A) and q-q plot (B) of GWAS results using the Nassar and Huhn S3 statistic as the model phenotype.


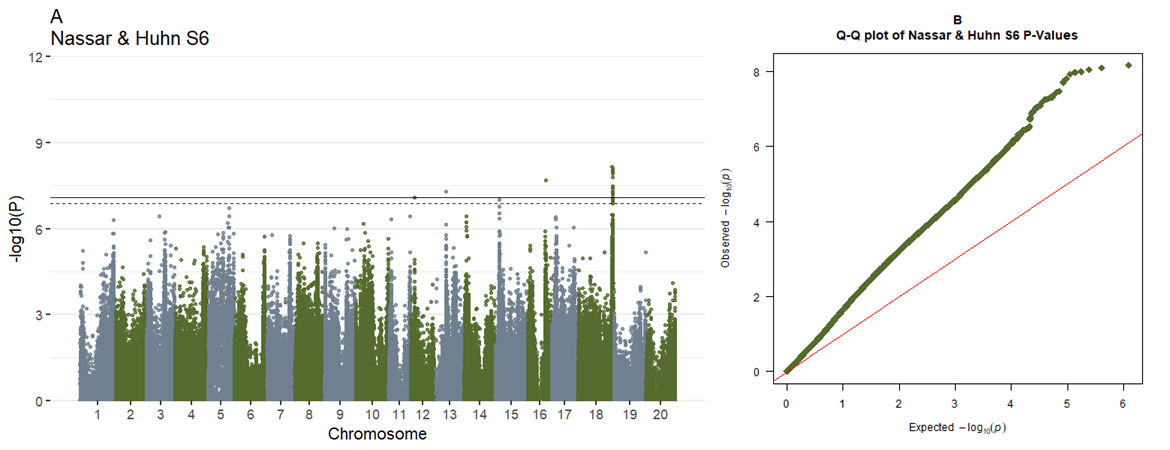


Supplementary Figure 13: Manhattan plot (A) and q-q plot (B) of GWAS results using the Nassar and Huhn S6 statistic as the model phenotype.


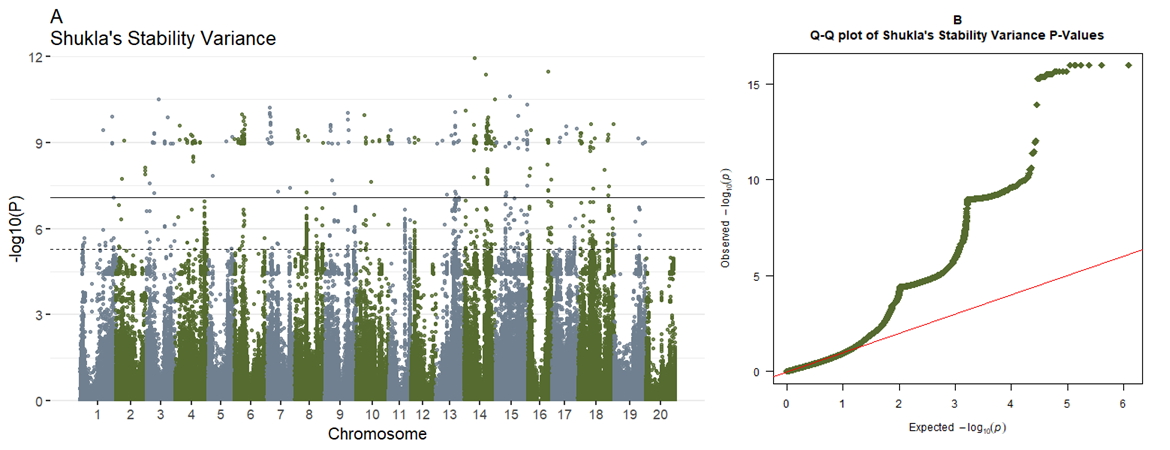


Supplementary Figure 14: Manhattan plot (A) and q-q plot (B) of GWAS results using Shukla’s Stability Variance as the model phenotype.


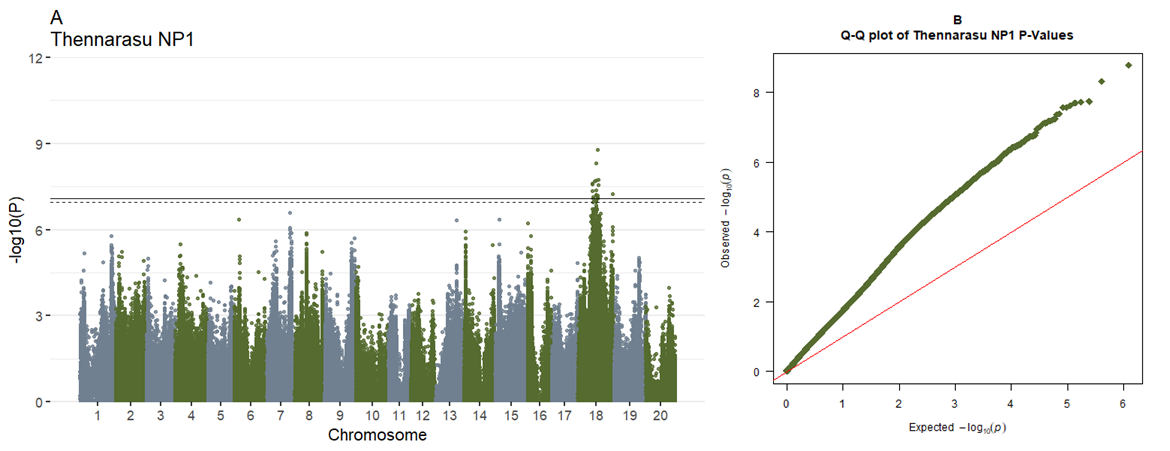


Supplementary Figure 15: Manhattan plot (A) and q-q plot (B) of GWAS results using the Thennarasu NP1 statistic as the model phenotype.


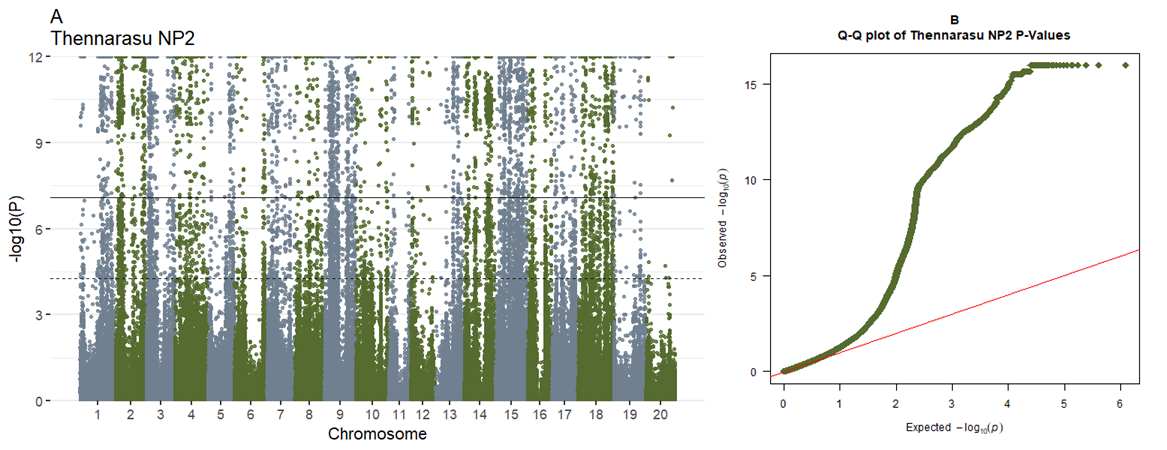


Supplementary Figure 16: Manhattan plot (A) and q-q plot (B) of GWAS results using the Thennarasu NP2 statistic as the model phenotype.


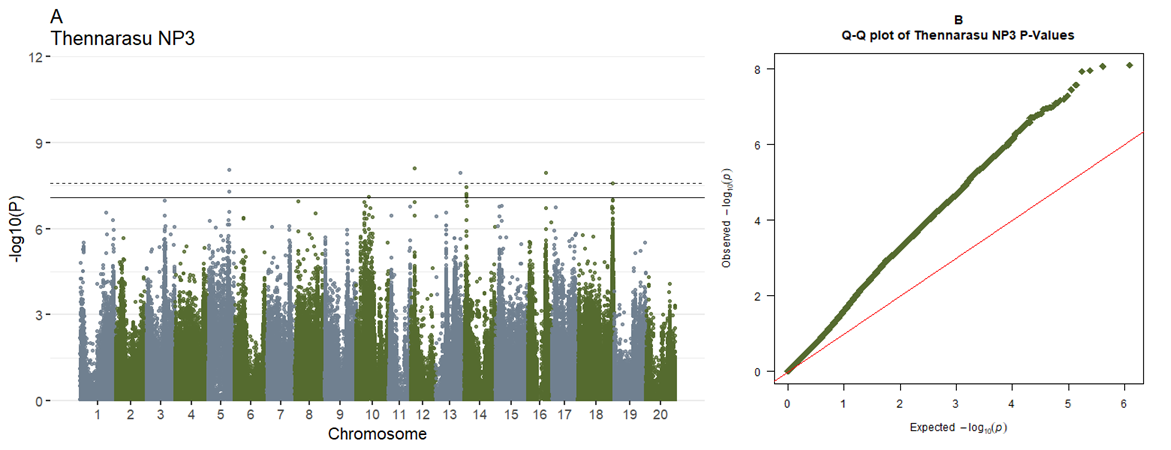


Supplementary Figure 17: Manhattan plot (A) and q-q plot (B) of GWAS results using the Thennarasu NP3 statistic as the model phenotype.


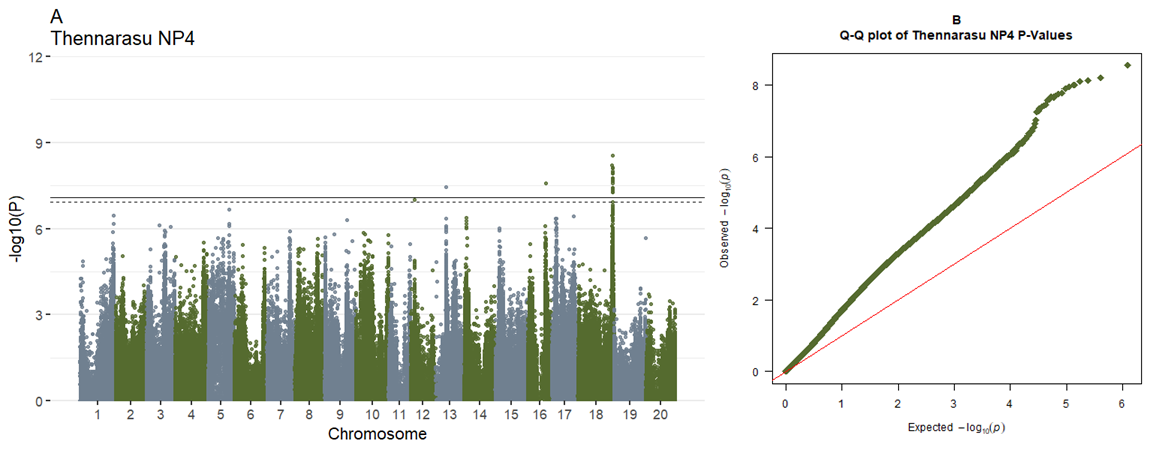


Supplementary Figure 18: Manhattan plot (A) and q-q plot (B) of GWAS results using the Thennarasu NP4 statistic as the model phenotype.


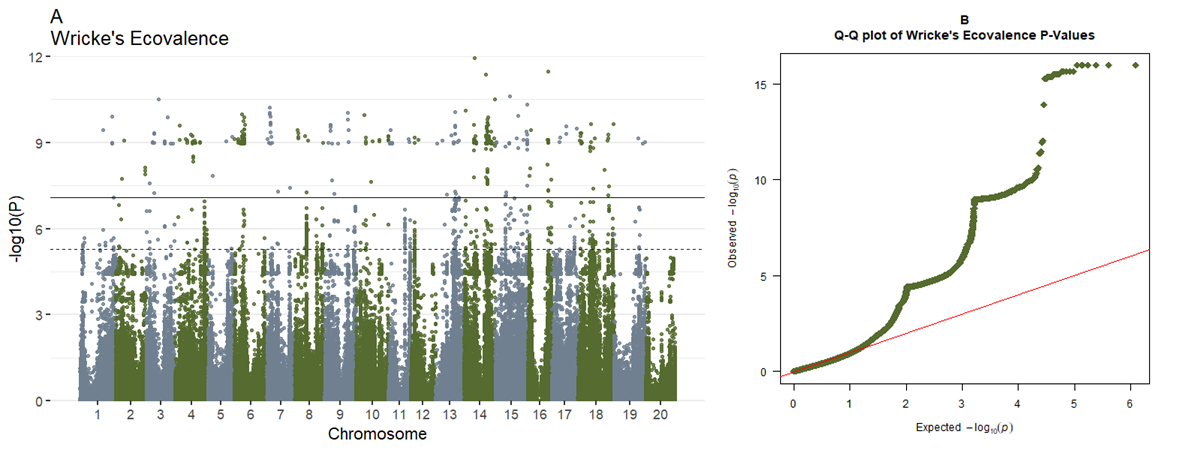


Supplementary Figure 19: Manhattan plot (A) and q-q plot (B) of GWAS results using Wricke’s Ecovalence as the model phenotype.


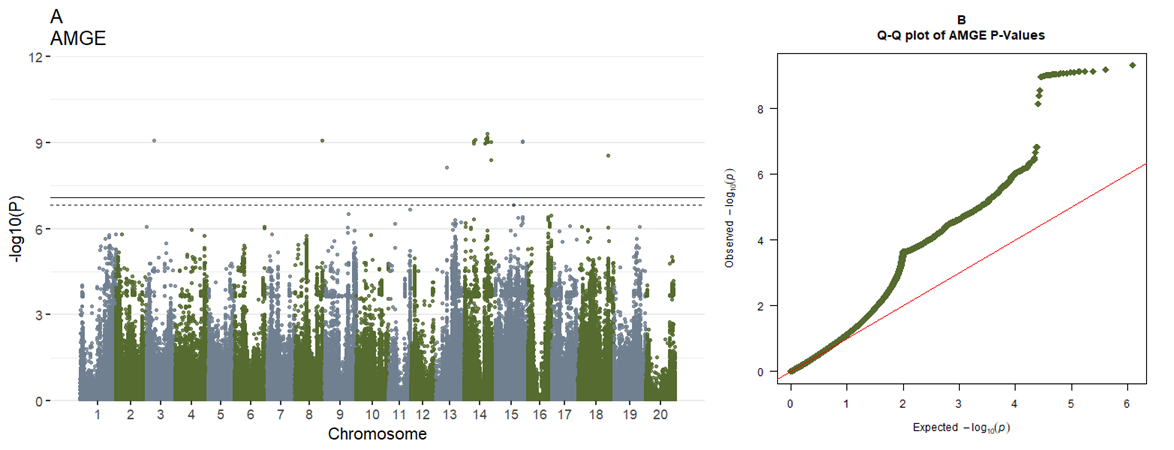


Supplementary Figure 20: Manhattan plot (A) and q-q plot (B) of GWAS results using the sum across environments of GEI modeled by AMMI as the model phenotype.


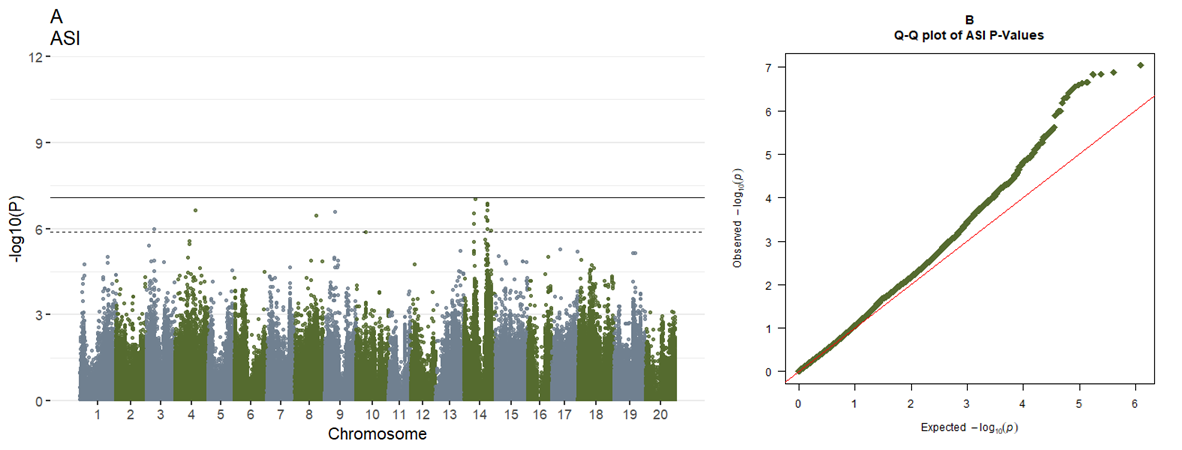


Supplementary Figure 21: Manhattan plot (A) and q-q plot (B) of GWAS results using the AMMI stability value as the model phenotype.


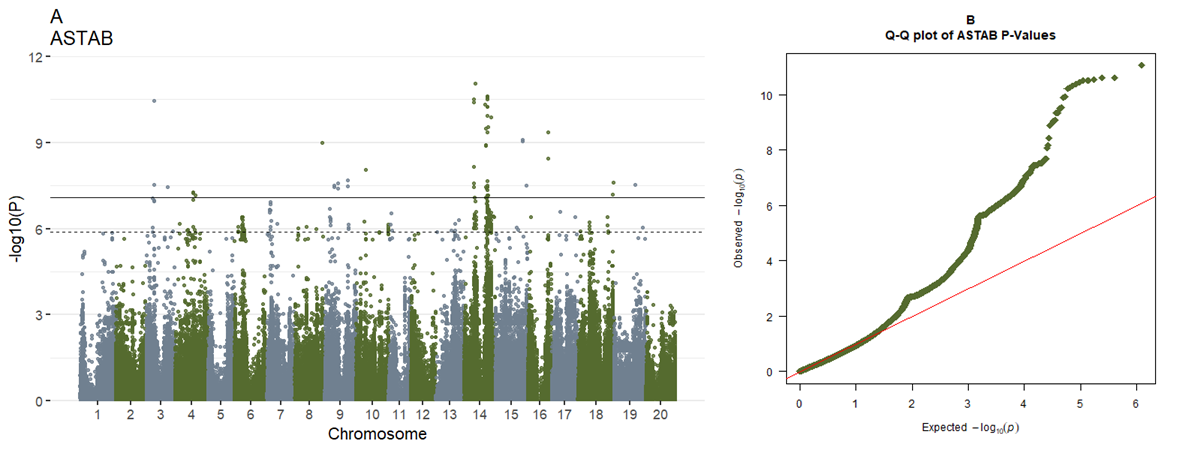


Supplementary Figure 22: Manhattan plot (A) and q-q plot (B) of GWAS results using the AMMI based stability parameter as the model phenotype.


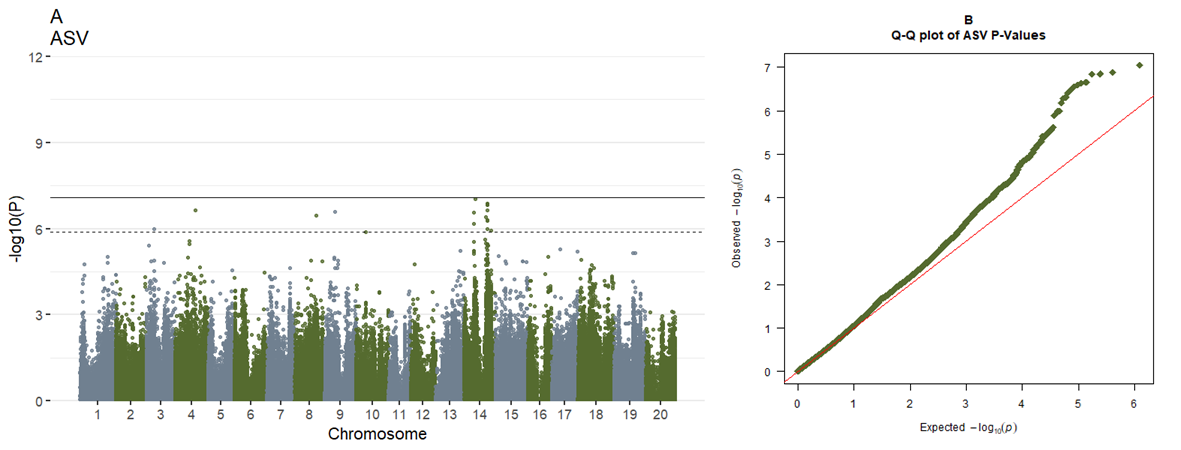


Supplementary Figure 23: Manhattan plot (A) and q-q plot (B) of GWAS results using the AMMI stability value as the model phenotype.


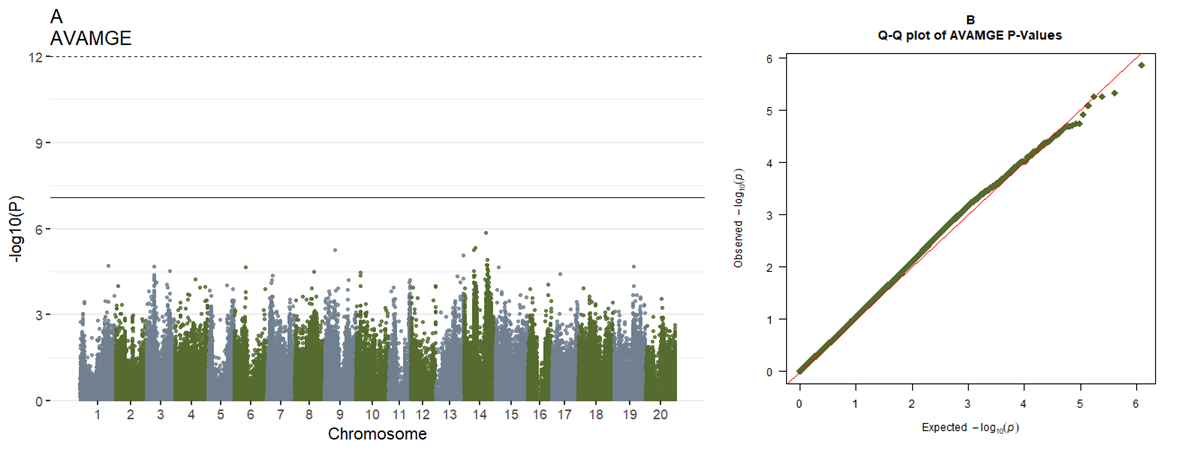


Supplementary Figure 24:: Manhattan plot (A) and q-q plot (B) of GWAS results using the sum across environments of absolute value of GEI modelled by AMMI as the model phenotype.


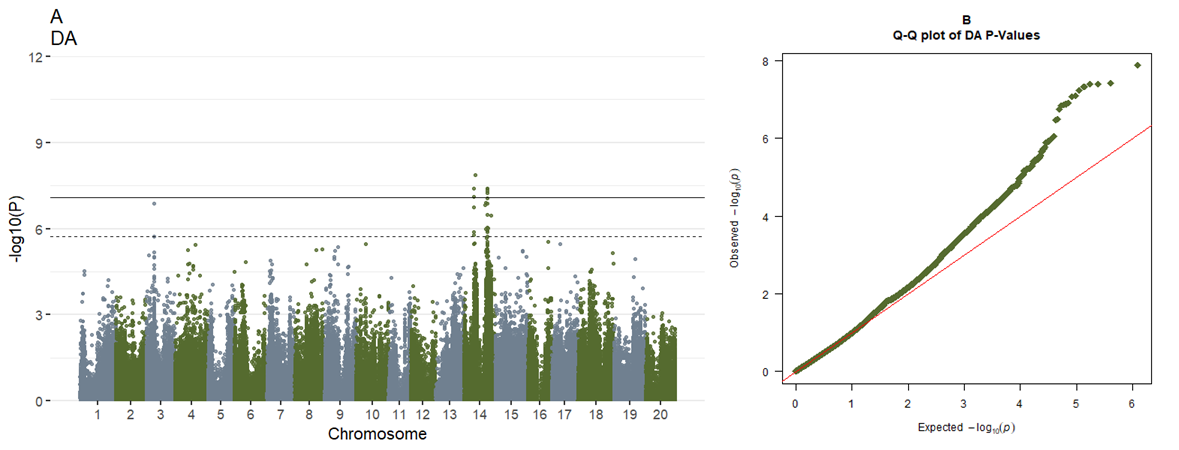


Supplementary Figure 25: Manhattan plot (A) and q-q plot (B) of GWAS results using Annicchiarico’s D parameter as the model phenotype.


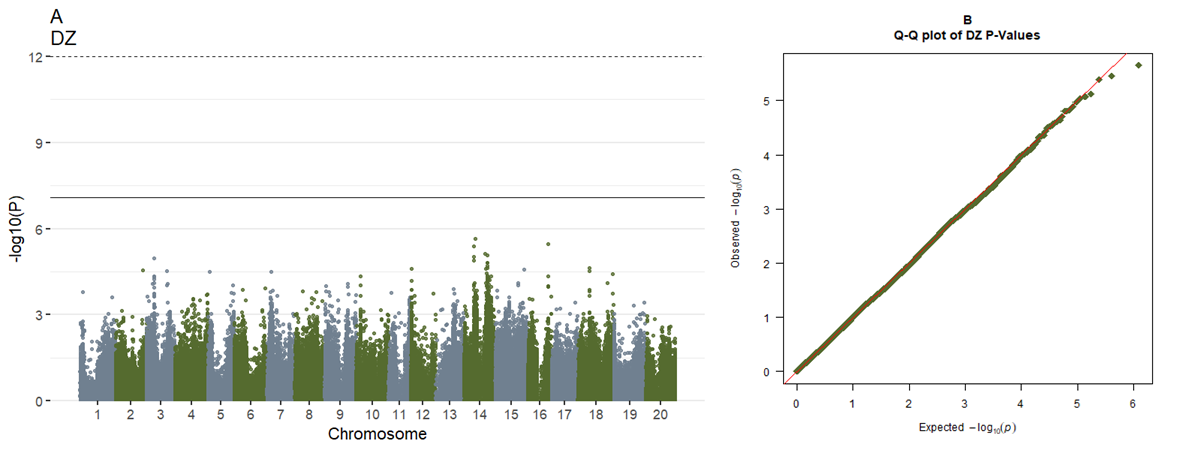


Supplementary Figure 26: Manhattan plot (A) and q-q plot (B) of GWAS results using Zhang’s D parameter as the model phenotype.


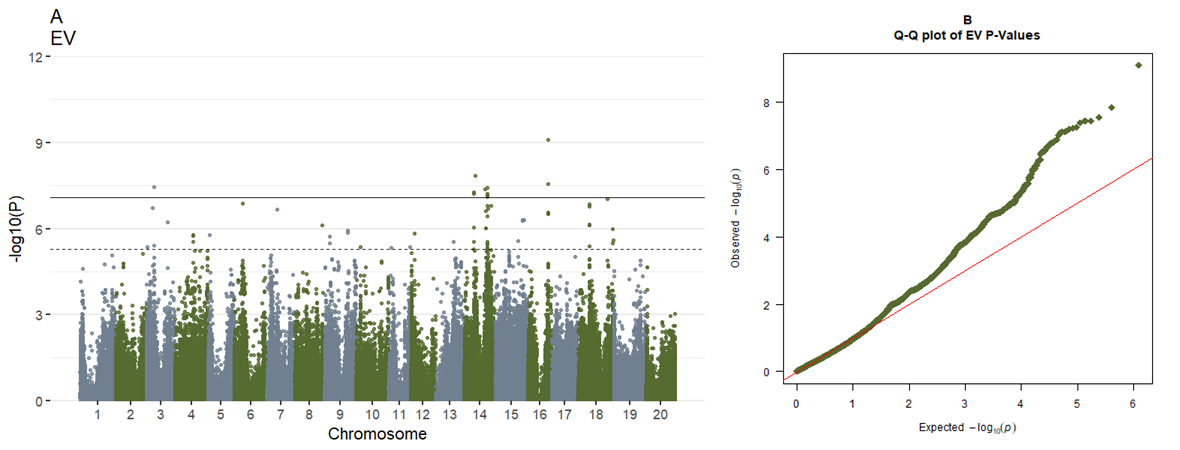


Supplementary Figure 27: Manhattan plot (A) and q-q plot (B) of GWAS results using the averages of the squared eigenvector values as the model phenotype.


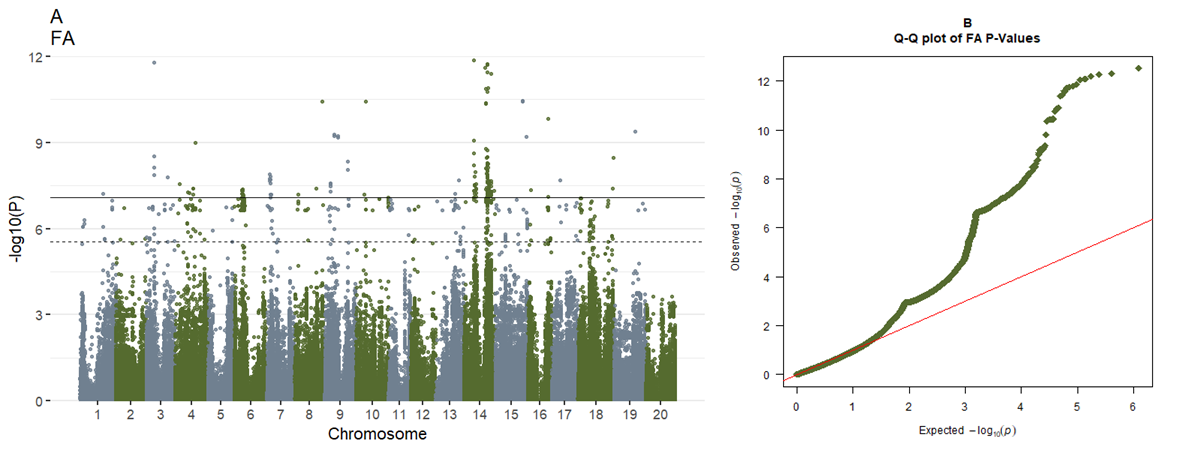


Supplementary Figure 28: Manhattan plot (A) and q-q plot (B) of GWAS results using the stability measure based on fitted AMMI model value as the model phenotype.


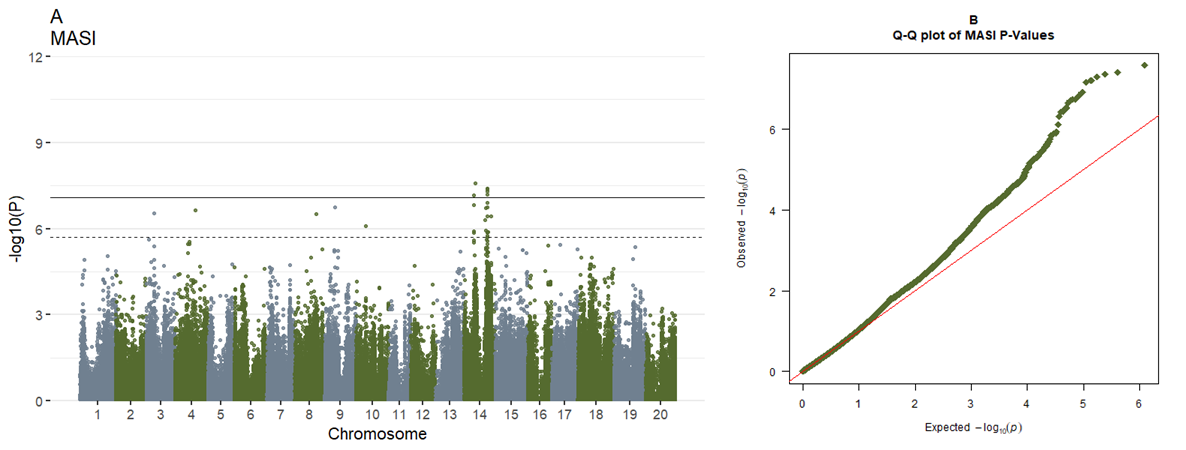


Supplementary Figure 29: Manhattan plot (A) and q-q plot (B) of GWAS results using the modified AMMI stability index as the model phenotype.


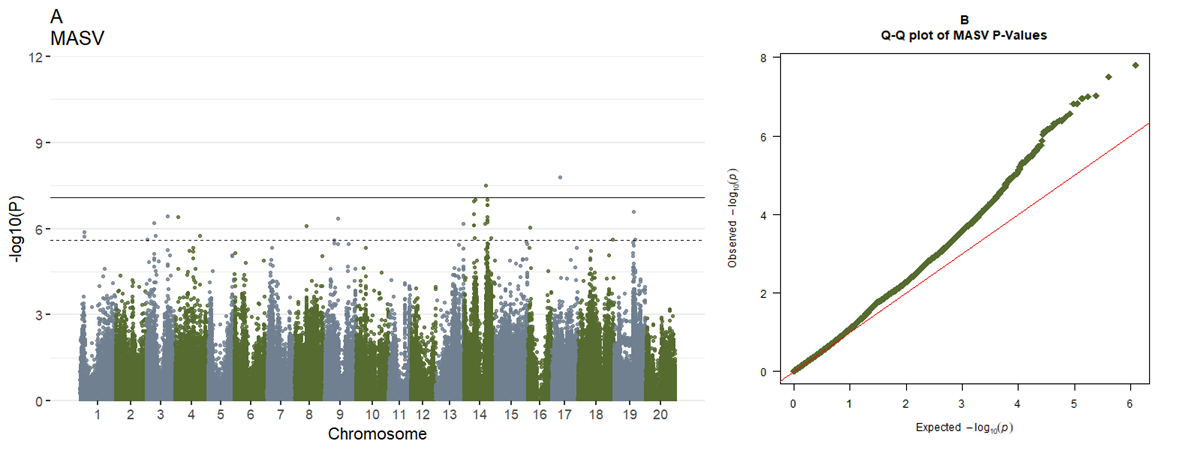


Supplementary Figure 30: Manhattan plot (A) and q-q plot (B) of GWAS results using the modified AMMI stability value as the model phenotype.


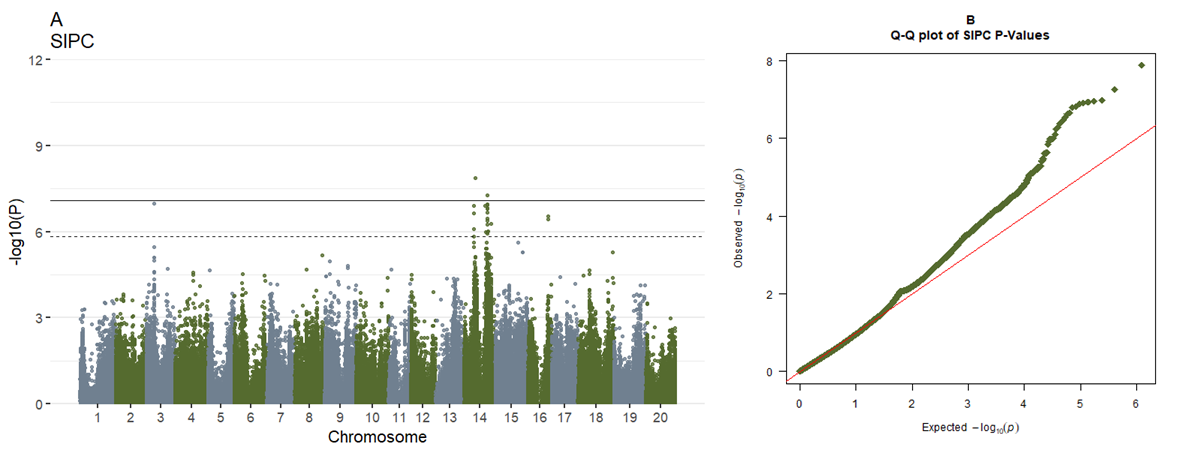


Supplementary Figure 31: Manhattan plot (A) and q-q plot (B) of GWAS results using the sums of the absolute value of the IPC scores as the model phenotype.


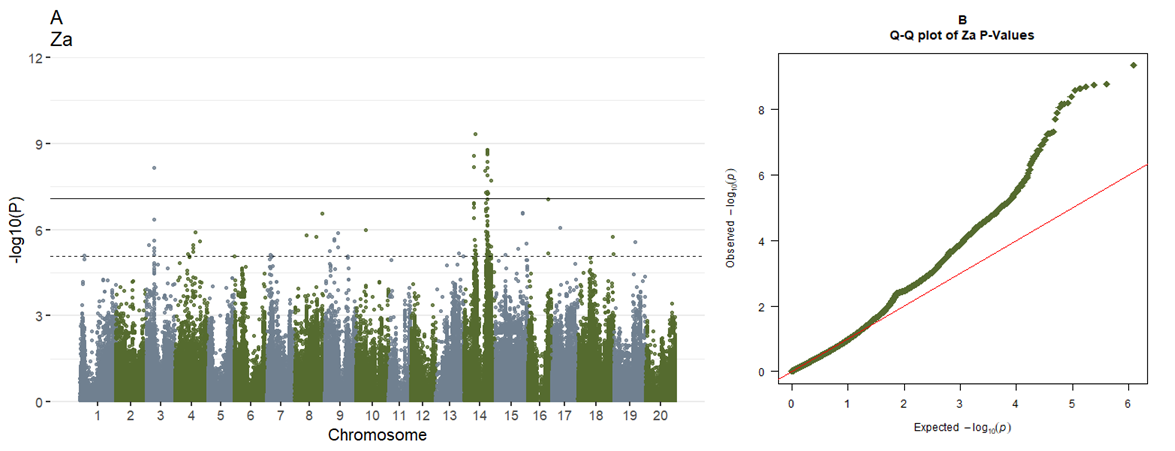


Supplementary Figure 32: Manhattan plot (A) and q-q plot (B) of GWAS results using the absolute value of the relative contribution of IPCs to the interaction as the model phenotype.


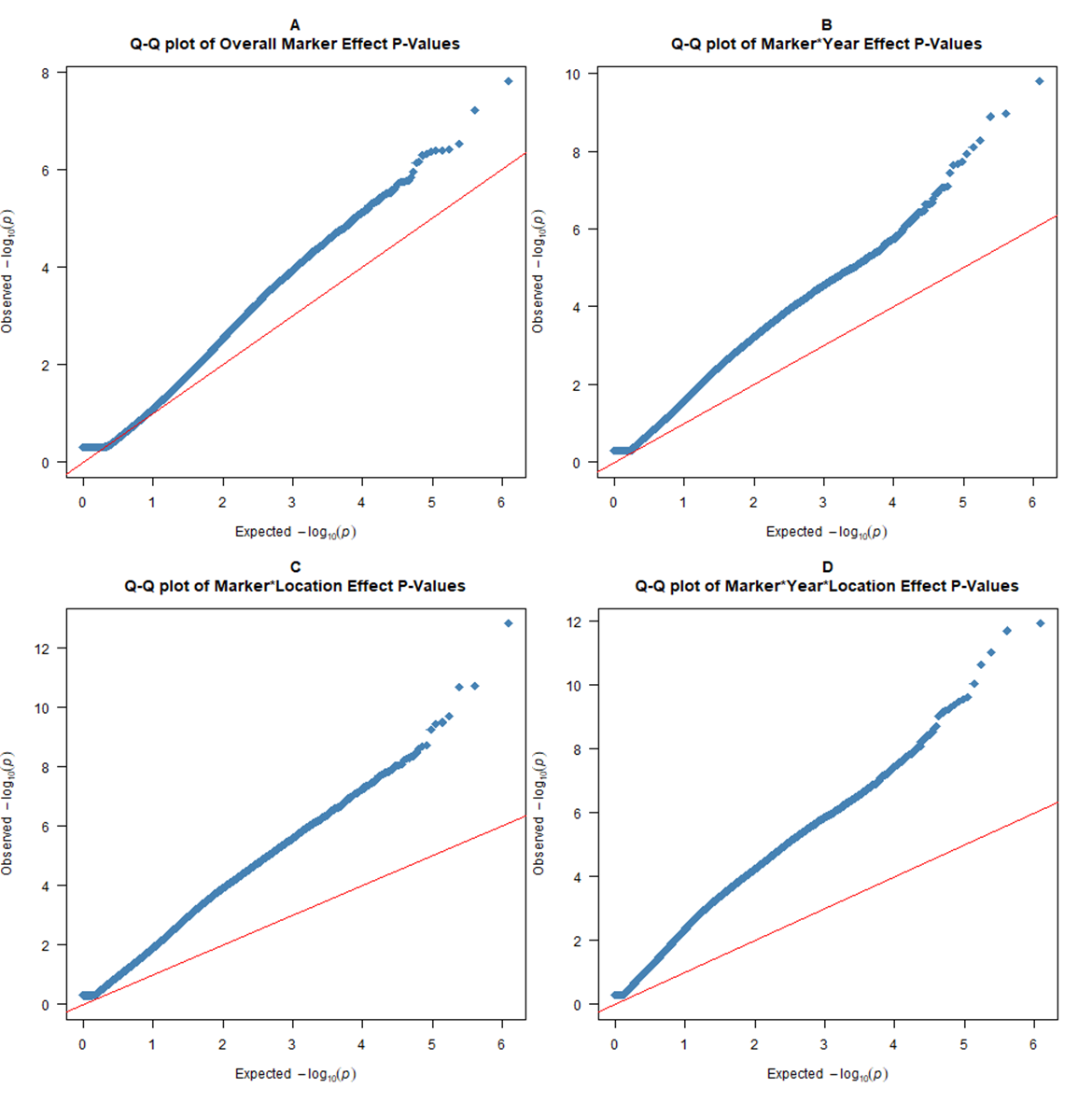


Supplementary Figure 33: Q-Q plots of each of the various marker interaction levels of explicitly modeling GxE interactions show that the results from more complex interactions are more inflated.


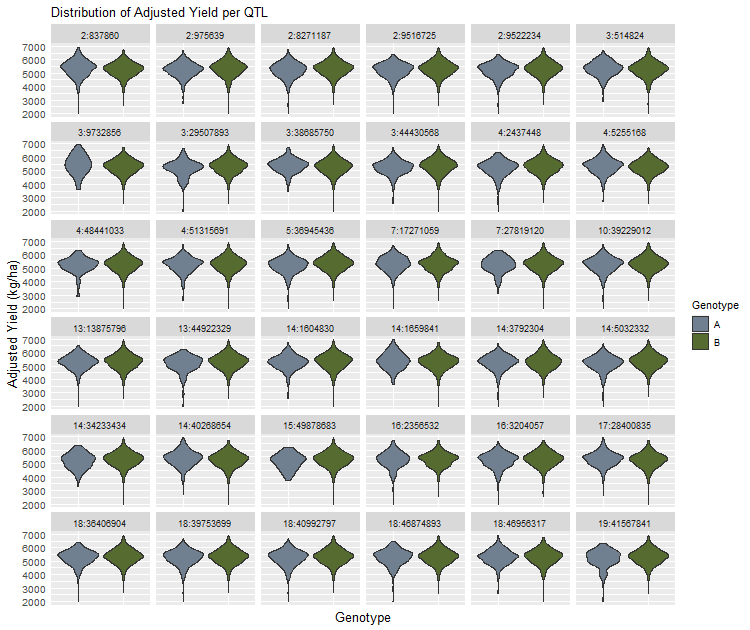


Supplementary Figure 34: Comparing the adjusted yield distributions of GxE QTL across environments shows that many QTL have no obviously advantageous allele when assessing the pooled data.

Supplementary Table 1: Five testing sites across eastern Nebraska were used to evaluate the yield performance of 213 soybean lines over three years for a combination of eleven unique environments. Lines were grouped according to maturity and assigned to field sites accordingly.

| **Location** | **Coordinates** | **Years** | **Maturity Groups** | **Lines Tested** | | | | |  |
| --- | --- | --- | --- | --- | --- | --- | --- | --- | --- |
|  |  |  |  | *MG I* | *early MG II* | *late MG II* | *MG III* | *Total* | |
| Cotesfield | 41.3581° N, 98.6338° W | 2018, 2019 | I, early II | 23 | 85 | x | x | 108 | |
| Lincoln | 40.8136° N, 96.7026° W | 2017, 2018, 2019 | late II, III | x | x | 64 | 41 | 105 | |
| Mead | 41.2286° N, 96.4892° W | 2018, 2019 | I, early II | 23 | 85 | x | x | 108 | |
| Phillips | 40.8980° N, 98.2132° W | 2017, 2018, 2019 | I, II, III | 23 | 85 | 64 | 41 | 213 | |
| Wymore | 40.1222° N, 96.6623° W | 2018 | late II, III | x | x | 64 | 41 | 105 | |

|  | Overall | Year | | | Location | | | | |
| --- | --- | --- | --- | --- | --- | --- | --- | --- | --- |
|  |  | ***2017*** | ***2018*** | ***2019*** | ***Cotesfield*** | ***Mead*** | ***Phillips*** | ***Lincoln*** | ***Wymore*** |
| ***Average*** | 4976.41 | 5204.80 | 4919.81 | 4859.99 | 5199.87 | 3995.49 | 5570.40 | 4491.35 | 4671.49 |
| ***Minimum*** | 2162.74 | 3227.30 | 2176.19 | 2162.74 | 2949.56 | 2176.19 | 2162.74 | 3327.51 | 3619.37 |
| ***Maximum*** | 7080.70 | 7080.70 | 6832.55 | 6353.06 | 6568.93 | 5419.64 | 7080.70 | 5672.50 | 5811.03 |
| ***Standard Deviation*** | 810.34 | 733.96 | 920.07 | 663.02 | 636.17 | 660.77 | 673.28 | 423.59 | 362.30 |

Supplementary Table 2: The overall grain yield ranged from 2162.74 to 7080.70 kg/ha with an average of 4976.41 kg/ha and standard deviation of 810.34 kg/ha. 2017 was the highest average yielding year and Phillips the highest yielding average location.

Supplementary Table 3: Univariate stability measures reflect a wide range of stability levels using multiple approaches.

|  | Thennarasu’s non-parametric statistics | | | | Huhn’s and Nassar and Huhn’s non-parametric statistics | | | | Wricke's Ecovalence | Shukla's Stability Variance | Deviation from regression | Regression coefficient | Coefficient of variance | GE variance component | Mean variance component | Kangs Rank Sum |
| --- | --- | --- | --- | --- | --- | --- | --- | --- | --- | --- | --- | --- | --- | --- | --- | --- |
|  | ***NP1*** | ***NP2*** | ***NP3*** | ***NP4*** | ***S1*** | ***S2*** | ***S3*** | ***S6*** | ***W_i_^2^*** | ***σ^2^_i_*** | ***S^2^_di_*** | ****b_i_*** | ***CVi*** | ***θ_(i)_*** | ***θi*** | ***KR*** |
| ***Average*** | 49.89 | 0.59 | 0.57 | 0.62 | 62.08 | 3436.49 | 347.33 | 5.10 | 287.32 | 28.86 | 72.59 | 0.54 | 12.19 | 28.86 | 28.93 | na |
| ***Minimum*** | 14.73 | 0.09 | 0.19 | 0.13 | 18.29 | 282.47 | 20.55 | 0.88 | 15.37 | 1.42 | 0.00 | 0.00 | 0.00 | 27.95 | 15.27 | na |
| ***Maximum*** | 88.64 | 6.56 | 1.52 | 1.46 | 117.16 | 11996.16 | 1189.88 | 13.28 | 2245.88 | 226.53 | 302.64 | 1.23 | 33.87 | 28.99 | 127.31 | na |
| ***Standard Deviation*** | 16.82 | 0.81 | 0.24 | 0.29 | 21.11 | 2150.63 | 251.54 | 2.60 | 315.48 | 31.84 | 43.95 | 0.21 | 4.04 | 0.15 | 15.85 | na |

Supplementary Table 4: Multivariate stability measures calculated from an AMMI model fit reflect a wide range of stability levels using multiple approaches.

|  | Sum Across Environments of GEI | AMMI Stability Index | AMMI Stability Value | AMMI Based Stability Parameter | Sum Across Environments of Absolute Value of GEI | Annicchiarico's D Parameter | Zhang's D Parameter | Averaged of the Squared Eigenvector Values | Stability Measure Based on Fitted AMMI Model | Modified AMMI Stability Index | Modified AMMI Stability Value | Sums of the Absolute Value of the IPC Scores | Absolute Value of the Relative Contribution of IPCs to the Interaction |
| --- | --- | --- | --- | --- | --- | --- | --- | --- | --- | --- | --- | --- | --- |
|  | ***AMGE*** | ***ASI*** | ***ASV*** | ***ASTAB*** | ***AVAMGE*** | ***DA*** | ***DZ*** | ***EV*** | ***FA*** | ***MASI*** | ***MASV*** | ***SIPC*** | ***Za*** |
| ***Average*** | 4.14E-18 | 3.13E-01 | 1.94E+00 | 1.74E+00 | 3.01E+01 | 1.36E+01 | 1.06E-01 | 4.61E-03 | 2.32E+02 | 3.30E-01 | 2.11E+00 | 1.81E+00 | 3.85E-02 |
| ***Minimum*** | -1.69E-13 | 2.24E-02 | 1.39E-01 | 8.25E-02 | 8.76E+00 | 3.48E+00 | 2.44E-02 | 1.99E-04 | 1.21E+01 | 5.99E-02 | 4.11E-01 | 4.65E-01 | 7.61E-03 |
| ***Maximum*** | 1.63E-13 | 1.18E+00 | 7.35E+00 | 9.81E+00 | 8.48E+01 | 3.96E+01 | 2.51E-01 | 2.11E-02 | 1.57E+03 | 1.19E+00 | 7.44E+00 | 4.56E+00 | 1.11E-01 |
| ***Standard Deviation*** | 5.63E-14 | 2.09E-01 | 1.30E+00 | 1.75E+00 | 1.56E+01 | 6.90E+00 | 5.06E-02 | 4.47E-03 | 2.53E+02 | 2.05E-01 | 1.27E+00 | 8.84E-01 | 2.05E-02 |
